# Epidemic and pandemic preparedness in the MENA region: A descriptive analysis of stakeholder distribution, patterns, and regional variation

**DOI:** 10.1371/journal.pgph.0007574

**Published:** 2026-11-06

**Authors:** Rim Alaeddine, Zahraa Chamseddine, Nour Zaouk, Zeinab Awad, Zahi Abdul Sater, Shadi Saleh

**Affiliations:** 1 Global Health Institute, American University of Beirut, Beirut, Lebanon; 2 Faculty of Health Sciences, American University of Beirut (AUB), Beirut, Lebanon; New York University Grossman School of Medicine, UNITED STATES OF AMERICA

## Abstract

Epidemic and pandemic preparedness relies on multi-sectoral coordination, a competent workforce, and well-defined preparedness functions. In the Middle East and North Africa (MENA) region, preparedness is shaped by diverse health system capacities, political contexts and recurrent crises. Despite recurrent outbreaks in the region, limited regional-level evidence exists on who is involved in preparedness and how different roles are distributed across preparedness domains, as well as countries and income settings. This cross-sectional stakeholder mapping study identified stakeholder roles, affiliations, and organizations involved in epidemic and pandemic preparedness across 22 MENA countries. Using publicly available online sources, stakeholder roles were systematically identified and categorized according to the European Centre for Disease Prevention and Control (ECDC) public health emergency preparedness framework. Records were analyzed by preparedness domain, workforce category, organizational affiliation, country, income level, and gender. A total of 3555 stakeholder roles were identified across the region. Roles were predominantly classified under coordination and communication (38.4%) and health services (32.0%), while emergency risk communication (4.1%) and policy development, adaptation, and implementation (6.6%) were the least represented domains. Emergency management roles constituted the largest workforce group (42.6%), followed by public health professionals (28.3%) and clinicians (19.4%). Significant variation was observed across country income levels, including differences in preparedness domains, workforce categories, organizational affiliation, and gender distribution. Records associated with women accounted for 31.5% of the total roles, with their representation varying across income groups. Preparedness-related roles were identified across the MENA region but vary in their distribution across domains, workforce categories, and countries. The greater representation of records in operational roles, alongside limited representation in policy development and emergency risk communication, highlights differences in the publicly identifiable preparedness landscape across functions. These findings provide a regional descriptive overview that can inform future validation, stakeholder engagement, and capacity-building efforts across the MENA region.

## Introduction

Epidemics and pandemics have been a recurring global health threat that has repeatedly overwhelmed health systems, disrupted essential services, and amplified existing inequities between countries [1,2]. Recent global health emergencies, most recently the COVID-19 pandemic, along with multiple ongoing outbreaks of infectious diseases demonstrated that effective preparedness extends beyond medical capabilities and includes resilient health systems, robust data surveillance and sharing, clear risk communication, and effective multi-sectoral and cross-border coordination [3–5]. Global frameworks, including the International Health Regulations (IHR), emphasize countries responsibility in developing core capacities for surveillance and response, as well as the importance of collaboration and cooperation throughout preparedness and response [6].

The Middle East and North Africa (MENA) region is characterized by diverse health system capacities, governance, and socio-political contexts. The region has experienced a range of outbreaks and disease threats in recent years, including endemic diseases like leishmaniasis, as well as emergent and re-emergent viral threats like Middle East Respiratory Syndrome (MERS) and COVID-19 [7]. Regional health security is further complicated by the ongoing crises, displacement, and conflict, increasing the likelihood of outbreaks across borders and amplifying the need for coordinated surveillance and response [8]. Key gaps range from unreliable and disintegrated data to lack of reporting and data sharing, limiting timely, evidence-based response and coordination [9]. During the COVID-19 pandemic, calls for access to timely, transparent, and reliable data in the region highlighted limitations in routine reporting and the challenges posed by lack of communication and governance [8]. In parallel, fragmented information systems, common in the region, can hinder rapid situational awareness and coordinated decision making [10,11].

Recent outbreaks in the MENA illustrate how cross-border threats intersect with fragmented institutional coordination and stakeholder networks. The emergence and circulation of the Middle East Respiratory Syndrome Coronavirus (MERS-CoV) highlighted the importance of rapid information exchange, coordinated surveillance, and clear institutional roles across countries [7]. Similarly, the cholera outbreak affecting conflict-affected countries in the region, particularly in Yemen, demonstrated how fragile systems, political instability, and fragmented governance and stakeholder engagement can undermine coordinated regional preparedness and response [12,13].These challenges are further magnified in settings experiencing protracted conflict and displacement, where multiple national, international, and humanitarian actors operate in parallel, without a clear map of roles and responsibilities across preparedness domains [14].

Effective preparedness requires multi-sectoral coordination, including clear roles, communication pathways, and governance mechanisms that enable rapid mobilization and coordinated action across sectors. Global evidence indicates that local actors’ engagement in preparedness and their coordination with higher governance levels is inconsistent, reflecting challenges in coordination and role clarity [15]. This is specifically relevant to the region, as evidence from MENA countries indicates that multi-sectoral collaboration during COVID-19 varied widely, with implementation shaped by governance arrangements and enabling environments, and with missed opportunities for sustained coordination across sectors [16].

This evidence highlights the persistent challenges in preparedness and response despite available resources as implementation can be undermined by insufficient information on relevant stakeholders, their roles, their sector, and relevance to preparedness. Mapping preparedness stakeholders provides a practical mechanism for understanding how preparedness systems function beyond theoretical frameworks. Identifying and mapping key actors can help streamline communication, facilitate cross-border engagement and knowledge exchange, and address documented surveillance challenges [17]. While stakeholder mapping has been applied in health systems research, existing efforts in the MENA region remain fragmented or country-specific, resulting in limited empirical visibility into how preparedness roles and responsibilities are distributed. To date, regional comparative evidence on stakeholder distribution across domains, workforce categories, organizational sectors, and country income settings in MENA remains incomplete. Gender representation is also an important dimension of health workforce composition. Although women comprise the majority of the global health and social care workforce, they remain substantially underrepresented in senior leadership and decision-making roles [2]. Recent work has further emphasized the importance of addressing gender and other workforce inequities within pandemic preparedness planning [18]

This study aims to systematically map and analyze stakeholder roles and affiliations identified in epidemic and pandemic preparedness across the MENA region, examining their distribution across preparedness domains, workforce categories, countries, income levels, and gender, and describing patterns and variation in the distribution of identified preparedness-related roles and gender representation across preparedness functions.

## Methods

This study employed a cross-sectional stakeholder mapping approach to identify and characterize stakeholders involved in epidemic and pandemic preparedness across the Middle East and North Africa (MENA) region. The mapping aimed to systematically document stakeholder roles, affiliations, and organizations engaged in preparedness related functions and to analyze their distribution across preparedness domains, workforce categories, country, income level, and gender. The study was designed as a descriptive mapping of preparedness-related roles and affiliations rather than an assessment of stakeholder influence, interests, or engagement. The study covered 22 countries in the MENA region. Stakeholder mapping was selected as a methodological approach given the fragmented and context specific nature of epidemics and pandemics, particularly in the MENA region. This descriptive study was conducted using publicly available online data sources to identify and map stakeholder roles across 22 countries in the Middle East and North Africa (MENA) region. Countries included were defined based on commonly used regional classifications in global health literature (e.g., WHO Eastern Mediterranean Region).

### Conceptual framework

The stakeholder mapping was guided by the European Centre for Disease Prevention and Control (ECDC) [19] public health emergency preparedness framework, which presents preparedness as a set of interrelated domains and capabilities required for effective prevention, detection, and response to public health emergencies. The framework highlights five key preparedness domains: 1. Detection and Assessment, 2. Policy development, adaptation, and implementation; 3. Health services; 4. Coordination and communication; 5. Emergency risk communication. Within the ECDC framework, Coordination and Communication refers to information sharing, incident management, and communication within the preparedness system, whereas Emergency Risk Communication focuses specifically on communication with the public. The framework was selected due to its ability to provide operationally defined preparedness functions across diverse health system contexts. For the purpose of this study, a stakeholder was defined as an individual publicly identified as holding a role, position, or responsibility relevant to one or more epidemic and pandemic preparedness domains within a MENA country.

### Search terms and data sources

#### Search strategy.

A structured online search strategy was developed to identify stakeholders involved in epidemic and pandemic preparedness. Search terms were initially derived from a narrative review of epidemic and pandemic preparedness literature and from the domains, capacities, and workforce categories outlined in the ECDC framework. Searches were conducted between 2023 and 2024 using publicly accessible online platforms, including general search engines (e.g., Google), official governmental and institutional websites, organizational reports, conference and webinar materials, and professional networking platforms such as LinkedIn. Searches were conducted primarily in English and Arabic, with consideration of commonly used terminology in the region.

A comprehensive list of search terms was developed to start the stakeholder identification process. Core search terms included combinations of the following keywords, adapted to each country context, for example: “Epidemic and pandemic preparedness stakeholders”, “Focal points for epidemic and pandemic preparedness”, “Health emergency preparedness stakeholders”. Emerging keywords identified during screening and data extraction were added to the initial keyword list, and searches were re-run using updated search strings. Each set of terms was paired with the name of the country of interest and searched using Google and other relevant online platforms. Search terms were iteratively refined based on data saturation and emerging terminology during the mapping process. The keywords used to guide stakeholder identification across preparedness functions, organizations, sectors, and professional roles are provided in S1 Table.

All search results were screened for relevance; only relevant search results were explored and probed for further findings. Initial stakeholder identification and screening were conducted by interns and research assistants. All search results were screened for relevance, and relevant sources were reviewed to determine whether identified individuals had a clearly described role or function related to epidemic and pandemic preparedness. Individuals were considered eligible when their publicly described roles were related to preparedness functions including surveillance and detection, response coordination, health services, policy, or risk communication. Despite standardization, the search was country-specific and adaptations were required given differences in the health systems in every country, the current situation of health emergency preparedness, the platforms used for dissemination of health information, the public availability of official documents and national health information, and the language usually utilized for dissemination of information in the country. Search results were screened iteratively within each country, and mapping continued until data saturation was reached. Data saturation was defined as the point at which repeated searches using the predefined and emerging search terms no longer identified substantially new stakeholder roles, organizations, or preparedness-related functions. Given differences in the availability of online information across countries, saturation was not based on a fixed number of search results or identified records.

#### Data collection.

Data was collected from publicly available online sources, including organizational websites, official reports, policy documents, webinars, conferences, workshops, formal and informal publications, and publicly accessible professional profiles. Social media platforms, particularly professional networking platforms such as LinkedIn, were also used when relevant to identify individuals actively engaged in preparedness-related roles. Targeted searches were conducted for organizations known to play key roles in epidemic and pandemic preparedness at national, regional, and international levels to enhance coverage and reduce omission of major actors. To ensure representation across preparedness capacities, stakeholder roles were systematically identified within each of the five preparedness domains outlined by the ECDC framework. Domain specific terms were used to expand the search within each country and to capture roles and functions involved in relevant capacities.

### Data collection process

Data were collected at both the stakeholder-role/affiliation and organizational levels, using mapping matrices developed in Microsoft Excel between 2023 and 2024.

For each stakeholder-role/affiliation record, data extracted and recorded in the database included the individual’s name, affiliated organization, country of residence, reported role or position, and gender, as reported or inferred from publicly available information. To enhance transparency and traceability, additional data were recorded for each entry, including the information source link, year of the source, and date of data extraction. Workforce categories were developed based on functional roles described in the ECDC preparedness framework and refined during data extraction to ensure alignment with operational preparedness functions. The workforce categories were used to group preparedness-related functions and were not intended to represent mutually exclusive professional identities. The analysis was based on stakeholder-role/affiliation records rather than unique individuals, allowing distinct roles and affiliations to be represented separately where applicable. Each record was classified according to the preparedness function described for that role. Following data extraction, the Research Associate and Program Manager reviewed the collected entries against the original sources to verify stakeholder identification, organizational affiliation, preparedness domain classification, and workforce classification. Any unclear or inconsistent entries were discussed and resolved during the validation process.

At the organizational level, data were extracted from publicly available sources, including the organization name, type of organization, country of operation, official website, available contact information (email address and phone number), and relevant departments or units involved in epidemic and pandemic preparedness, where applicable. The distinction between individual- and organizational-level mapping was intended to capture relationships between institutions and the roles identified within them.

### Statistical analysis

Data were analyzed using descriptive and inferential statistical methods. Categorical variables were summarized as frequencies and percentages to describe characteristics of stakeholder records, including gender, domain of action, workforce category, organizational affiliation, and country income classification.

For comparative analysis, record characteristics were stratified by country income level (low-income, lower-middle-income, upper-middle-income, and high-income countries), based on the World Bank's country income classification [20]. To assess differences in these characteristics across income levels, chi-square (χ²) tests were conducted. Statistical significance was defined as a two-sided p-value < 0.05.

Given the descriptive nature of the stakeholder mapping, no imputation was performed for missing data. Variables with incomplete data were excluded from specific analyses, and analyses were conducted using available data only.

A cross-tabulation table was used to present the distribution of workforce categories across preparedness domains, while geographic mapping was used to depict the spatial distribution of stakeholder roles across countries.

All statistical analyses were conducted using Stata/SE version 18 (StataCorp LLC, College Station, TX, USA), and visualizations were created using Flourish (Flourish Studio, London, UK).

### Ethics statement

This study relied exclusively on publicly available information and did not involve interaction with human participants or collection of non-public personal data. The broader “Epidemic and Pandemic Preparedness” project, which included desk-review-based identification and mapping of preparedness stakeholders, received exempt approval from the Institutional Review Board (ID: SBS-2022–0256) of the American University of Beirut. The present analysis was limited to publicly available professional information and involved no direct interaction with the individuals identified or access to non-public data.

## Results

### Stakeholder record characteristics

Table 1 summarizes demographic, professional, and organizational characteristics of the 3,555 stakeholder-role/affiliation records identified across 22 MENA countries. Overall, 68.52% of records were associated with men (n = 2,436), while 31.48% were associated with women (n = 1,119). The largest proportion of records was classified within Coordination and Communication domain (38.5%; n = 1367), followed by health services (32.01%; n = 1,138) and detection and assessment activities (18.82%; n = 669). Policy development and emergency risk communication were the least represented domains, accounting for 6.64% (*n* = 236) and 4.08% (*n* = 145) of records, respectively. In terms of country income classification, nearly half of the stakeholder roles were from lower-middle-income countries (45.43%; n = 1,615), followed by high-income countries (28.66%; n = 1,019). Records from low-income countries constituted 14.32% (n = 509) of the sample, while those from upper-middle-income countries represented 11.59% (n = 412). Regarding workforce categories, emergency managers formed the largest group (42.56%; n = 1,513), followed by public health professionals (28.27%; n = 1,005) and clinicians (19.41%; n = 690). Smaller proportions of records were classified as policymakers (4.08%; n = 145), public health communicators (3.77%; n = 134), and civil society leaders (1.91%; n = 68). Among the organizations identified through the mapping process, public sector organizations accounted for the largest share (39.85%; n = 371), while private sector organizations accounted for 27.39% (n = 255). Local non-governmental organizations represented 15.57% (n = 145), regional NGOs 11.17% (n = 104), and research centers constituted 6.02% (n = 56) of total organizations.

**Table 1:** Overview of Stakeholder-Role Record Characteristics (For conciseness in tables and figures, some preparedness domain names are presented in abbreviated form, corresponding to the full European Centre for Disease Prevention and Control framework domains as described in the Methodology section).

Variables	N	%
**Stakeholder Characteristics (n=3555)**		
**Gender**		
Female	1,119	31.48
Male	2,436	68.52
**Preparedness Domain**		
Coordination and Communication	1,367	38.45
Detection and Assessment	669	18.82
Emergency Risk Communication	145	4.08
Health Services	1,138	32.01
Policy Development	236	6.64
**Income Level**		
High-income	1019	28.66
Upper-middle-income	412	11.59
Lower-middle-income	1615	45.43
Low-income	509	14.32
**Workforce Category**		
Civil Society Leader	68	1.91
Clinician	690	19.41
Emergency Manager	1,513	42.56
Policymaker	145	4.08
Public Health Communicator	134	3.77
Public Health Professional	1,005	28.27
**Organization Type**		
Public Sector	371	39.85
Private Sector	255	27.39
Regional NGO	104	11.17
Local NGO	145	15.57
Research Center	56	6.02

### Geographic distribution of stakeholder roles

Geographic distribution analysis showed substantial variation in the number of identified roles across MENA countries (Fig 1). The largest absolute numbers of records were identified in the United Arab Emirates (n = 360), Lebanon (n = 341), Palestine (n = 326), and Egypt (n = 324), while Mauritania and Syria had the lowest absolute counts (n = 42 each). To account for differences in population size, identified stakeholder-role records were also standardized per 100,000 population (S2 Table). The highest population-standardized values were observed in Bahrain (11.75 records per 100,000 population), Palestine (5.83), Lebanon (5.83), and Comoros (5.78). Granular country-level data, including exact counts, are provided in the form of country cards (S1 Fig).

**Fig 1:**
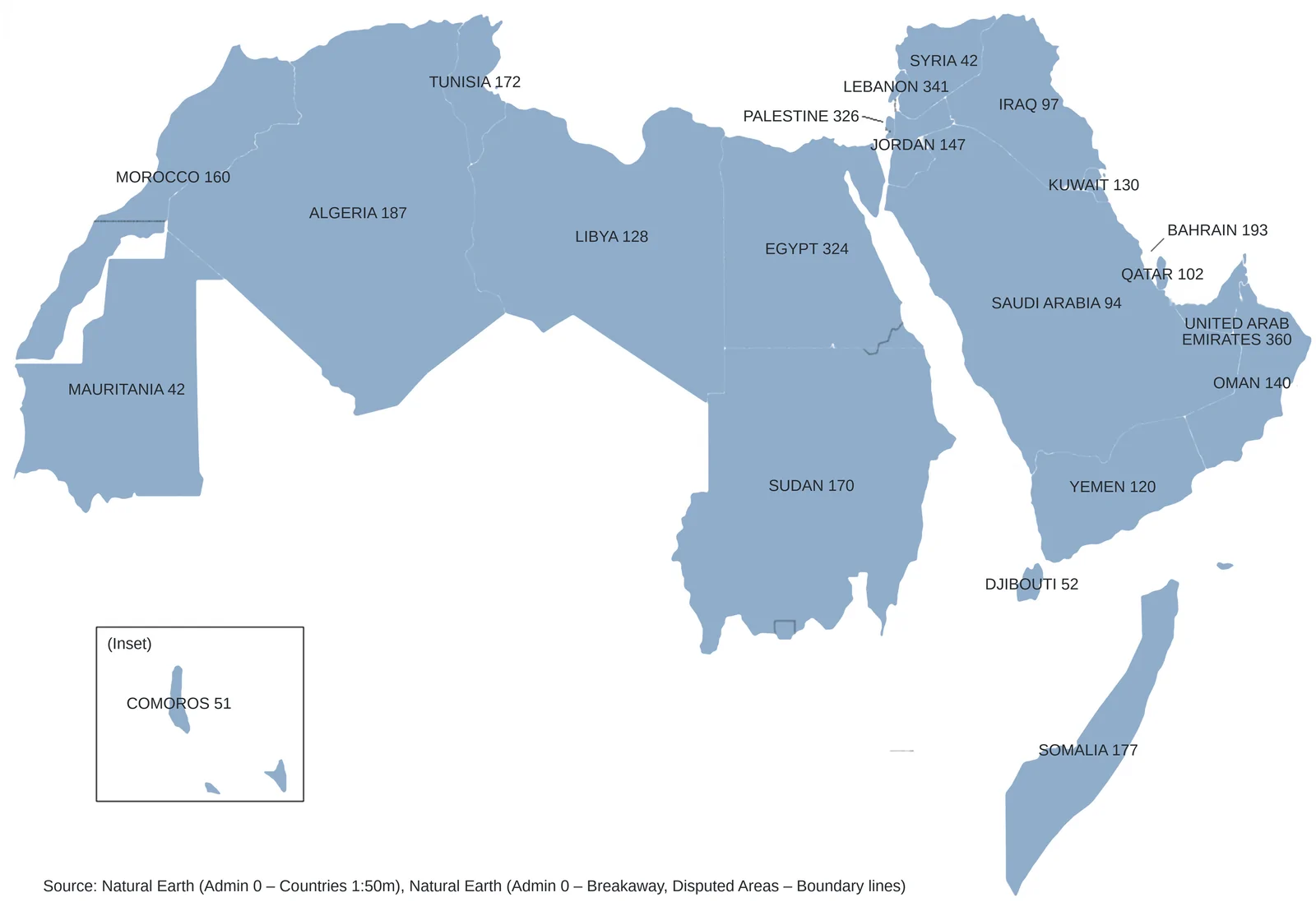
Geographic distribution of identified stakeholder roles across MENA countries. Labels indicate absolute numbers of records identified through stakeholder mapping. Comoros is displayed in an inset. Country boundaries are based on Natural Earth administrative boundary data.

### Workforce distribution across preparedness domains

Table 2 presents the distribution of workforce categories of stakeholder roles across the five preparedness domains. The results indicated that emergency managers constituted the largest workforce group (n = 1,513) and were predominantly engaged in coordination and communication activities, in addition to health services. Among records classified as Public Health Professional (n = 1,005), most were assigned to Detection and Assessment (n = 578), followed by Health Services (n = 245) and Coordination and Communication (n = 163). Records classified as Clinician (n = 690) were predominantly assigned to Health Services (n = 625), with relatively few in Detection and Assessment (n = 57). While other workforce groups demonstrated multisectoral engagement, policymakers and public health communicators were primarily involved in policy development and emergency risk communication, respectively. Civil society leaders represented the smallest workforce group (n = 68) and demonstrated very limited engagement across all domains.

**Table 2:** Distribution of workforce categories across preparedness domains (N = 3,555).

Workforce category	Coordination & Communication	Detection & Assessment	Emergency Risk Communication	Health Services	Policy Development, Adaptation & Implementation	Total
Civil Society Leader	56	0	3	8	1	68
Clinician	5	57	2	625	1	690
Emergency Manager	1,132	34	5	259	83	1,513
Policymaker	6	0	1	0	138	145
Public Health Communicator	5	0	128	1	0	134
Public Health Professional	163	578	6	245	13	1,005
**Total**	**1,367**	**669**	**145**	**1,138**	**236**	**3,555**

### Stakeholder characteristics by country income level

Table 3 summarizes the characteristics of stakeholder-role records and their corresponding organizational affiliations stratified by country income level. Significant differences were observed across all examined characteristics, including gender, pandemic preparedness domain, workforce category, and organization type (all p < 0.001). Across income groups, records associated with men predominated in all settings, particularly in low-income countries (78.19%) and upper-middle-income countries (76.94%). Records associated with women accounted for 21.81% in low-income countries, 31.52% in lower-middle-income countries, 23.06% in upper-middle-income countries, and 39.65% in high-income countries. Coordination and Communication represented the largest preparedness domain across all four income groups, accounting for 35.36% in low-income, 39.32% in lower-middle-income, 41.50% in upper-middle-income, and 37.39% in high-income countries. Health Services was the second most represented domain in lower-middle-income (35.54%), upper-middle-income (24.03%), and high-income countries (35.33%), while Detection and Assessment ranked second in low-income countries (31.43%). Detection and assessment domain was more evenly distributed but showed relatively higher representation in low-income countries (31.43%). Policy development domain was more prevalent in upper-middle-income countries (10.19%) compared with other income groups (p < 0.001). Workforce composition varied significantly by income level. Emergency managers constituted the largest workforce group in lower-middle-income (46.38%), upper-middle-income (44.66%), and high-income countries (44.36%), whereas public health professionals constituted the largest group in low-income countries (51.28%). Clinicians were most strongly represented in high-income countries (21.98%). Policymakers and civil society leaders accounted for small proportions across all income groups. Organizational affiliation also differed by income classification.

**Table 3:** Characteristics of Stakeholder-role records and Organizational affiliations by country income level.

		LIC	LMIC	UMIC	HIC	
	n	%	%	%	%	*P* value
**Gender**						<0.001*
Female	**1,119**	21.81	31.52	23.06	39.65	
Male	**2,436**	78.19	68.48	76.94	60.35	
**Domain**						<0.001*
Coordination and Communication	**1,367**	35.36	39.32	41.50	37.39	
Detection and Assessment	**669**	31.43	14.74	19.17	18.84	
Emergency Risk Communication	**145**	3.93	4.27	5.10	3.43	
Health Services	**1,138**	20.63	35.54	24.03	35.33	
Policy Development	**236**	8.64	6.13	10.19	5.00	
**Workforce Category**						<0.001*
Civil Society Leader	**68**	2.36	2.29	2.43	0.88	
Clinician	**690**	9.63	20.62	20.39	21.98	
Emergency Manager	**1,513**	25.15	46.38	44.66	44.36	
Policymaker	**145**	7.47	3.22	3.88	3.83	
Public Health Communicator	**134**	4.13	3.90	4.85	2.94	
Public Health Professional	**1,005**	51.28	23.59	23.79	26.01	
**Organization Type**						<0.001*
Research Center	**56**	1.68	7.76	11.19	2.08	
Public Sector	**371**	26.89	30.14	61.19	52.08	
Regional NGO	**104**	15.97	11.42	10.45	8.75	
Local NGOs	**145**	48.74	15.30	8.21	3.75	
Private Sector	**255**	6.72	35.39	8.96	33.33	

^*^Indicates statistical significance at *p* < 0.05.

## Discussion

This study provides a regional mapping and descriptive overview of the epidemic and pandemic preparedness stakeholder landscape across the MENA region, responding to persistent gaps in information on who is involved in preparedness, how roles are distributed, and where potential weaknesses may exist. By mapping stakeholder roles and affiliations across 22 countries, this analysis characterizes the structure of preparedness systems. The findings indicate that while preparedness-related activity is present across the region, its distribution reveals marked variation in the representation of preparedness functions and workforce categories, particularly in relation to strategic functions, coordination, and leadership.

### Distribution of stakeholder records across preparedness domains and workforce

The concentration of stakeholder roles within coordination and communication and health service domains suggests that the publicly identifiable preparedness landscape in the MENA region is more strongly represented in operational response functions. This is consistent with the region’s recent outbreaks and protracted crises, which have placed major emphasis on immediate response, operational coordination, and the importance of continuity of service delivery. Previous outbreaks, like MERS-CoV and COVID-19, clearly highlighted the importance of rapid coordination mechanisms and functional health services to manage increased pressure on health systems [7,14]. Coordination and Communication emerged as the most represented preparedness domain, closely followed by Health Services. However, preparedness frameworks conceptualize preparedness as a multidimensional process that extends beyond coordination and service delivery, to include surveillance, policy, governance and communication capacities [5]. The observed greater representation of records in operational domains raises questions about the balance between immediate response capacity and the longer-term, more strategic preparedness functions needed to prevent, detect, and manage future threats effectively. These patterns may reflect the broader political and institutional context of the MENA region, where preparedness systems are frequently shaped by recurrent crises, humanitarian responses, and externally driven interventions This pattern may reflect greater emphasis on operational needs than on longer-term system and policy functions.

Emergency risk communication emerged as the least represented preparedness domain (4.08%), indicating comparatively limited representation within the identified stakeholder landscape. Despite extensive evidence from recent outbreaks, including those in the MENA region, demonstrating the central role of communication in shaping public trust, ensuring adherence to public health measures, and fostering community engagement [8,16], this domain remains comparatively less represented among identified records. During the COVID-19 pandemic, weaknesses in communication systems were associated with misinformation, inconsistent messaging, and erosion of trust, even in settings with robust technical and clinical response capacities [21]. The limited visibility of emergency risk communication roles may reflect communication functions that are less institutionally visible or that become more prominent during periods of crisis rather than being continuously represented within preparedness systems across much of the region. Similarly, Policy Development roles comprised only 6.64% of the total, indicating a potential underemphasis on the strategic development and implementation of sustainable preparedness policies. Together, these patterns require further assessment of whether lower representation of identified roles reflects workforce composition, institutional priorities, differences in online visibility, or actual gaps in the organization of these functions.

Country level observation further indicates that among identified stakeholder roles, representation in several settings was concentrated within a single dominant domain, most commonly coordination or emergency management, with minimal representation across other domains and workforce categories. In countries such as Djibouti and Mauritania, where fewer records were identified and were largely clustered within coordination or emergency response roles, the identified stakeholder landscape appears more strongly centered around incident management. Rather than reflecting a balanced distribution across preparedness domains, this distribution suggests greater representation of acute-response functions than of anticipatory planning, policy development, and sustained communication capacity. While such focus may facilitate rapid operational response during crises, it also risks constraining preparedness to be reactive, limiting sustained investment, policy development, and crucial inter-crisis coordination [5,22]. Lower numbers of publicly identifiable roles may also reflect limited online visibility, documentation practices, or reporting differences across countries, highlighting limitations in data availability and transparency that may influence stakeholder identification.

Workforce distribution further supports the preparedness landscape’s operational orientation. Emergency Manager was the largest workforce category (42.56%), followed by Public Health Professionals (28.27%) and Clinicians (19.41%), illustrating the greater representation of operational and response-oriented roles within the mapped workforce. While these roles are essential during acute response phases, literature increasingly emphasizes the importance of embedding preparedness within robust governance structures, policy processes, and strategic planning mechanisms that persist beyond crisis periods [23,24]. Table 2 illustrates that emergency managers and public health professionals are predominantly engaged in coordination, communication, and health services, while clinicians are concentrated within health services. The lower representation of roles linked to policy and strategic functions raises questions about how lessons from response are translated into policy, enduring frameworks, continuous training, and longer-term institutional development.

At the country level, several countries were characterized by a comparatively small number of identified stakeholder roles across multiple preparedness domains. This may reflect lower visible representation, although further country-level validation would be needed to determine whether this corresponds to a genuinely narrower institutional or workforce base. Where a limited workforce base is confirmed through country-level validation, dependence on a smaller number of actors could have implications for continuity during staff turnover or political disruption. In MENA, where health systems in several countries are shaped by conflict, political instability, and resource constraints, preparedness systems with limited domain and workforce range may face additional challenges in sustaining capacity and developing preparedness beyond outbreaks [16,23].

### Organizational affiliation and income-level disparities

Variation in organizational affiliation across income groups highlights how broader political and economic contexts shape preparedness structures. Table 3 presents significant variations in organization distribution based on income levels. Lower-income and conflict-affected settings showed greater representation of non-governmental institutions. For instance, NGOs accounted for a substantial 48.74% of organizational affiliations in Low-Income Countries, in stark contrast to 3.75% in High-Income Countries. This pattern aligns with existing evidence from fragile settings, where preparedness and response functions are frequently distributed across systems [12,25]. This particularly overlaps with the presence of conflict and instability. Countries experiencing protracted conflict or humanitarian crises, such as Yemen and Syria, tend to exhibit smaller, more fragmented preparedness ecosystems and greater reliance on international and humanitarian organizations. While such institutions can support short-term response and fill critical gaps during crises, greater reliance on multiple external and humanitarian actors may also create additional coordination and accountability challenges and may complicate the institutionalization of preparedness within national systems. In contrast, higher-income contexts appeared to rely more heavily on public sector institutions, with Public Sector affiliations dominating in HICs at 52.08%, suggesting greater state centrality in preparedness functions. Conversely, Private Sector representation was notably higher in Lower-Middle Income Countries at 35.39% and HICs at 33.33%, indicating varied engagement of the private sector across the region.

### Regional variation in identified stakeholder roles

Beyond income-level categorization, the country-level analysis of stakeholder distribution reveals substantial variation in the number of identified stakeholder roles across the region. Some countries, such as the United Arab Emirates, Egypt, and Lebanon, had comparatively large absolute numbers of identified records spanning multiple domains and sectors, while others, including Djibouti and Mauritania, had lower absolute numbers of identified records. However, these absolute counts partly reflect differences in population size. When standardized by population, higher numbers of identified stakeholder roles per 100,000 population were observed in Bahrain, Palestine, Lebanon, and Comoros. These standardized values should be interpreted cautiously, as they reflect records identified through the mapping process rather than the number of unique individuals or the size or adequacy of each country’s preparedness workforce.

The observed cross-country variation does not necessarily reflect the total number of individuals within each country’s preparedness workforce. Observed differences may reflect limited institutionalization of preparedness functions, reliance on informal networks, or weak documentation and transparency practices. Limited visibility can place significant constraints on preparedness by limiting coordination, information sharing, and cross-border collaboration. This is especially relevant in the context of transnational health threats, where effective preparedness depends on clear lines of communication and collaboration across countries, as emphasized in the International Health Regulations and related global frameworks [5,26]. Furthermore, evidence from the MENA region suggests that multi-sectoral collaboration during COVID-19 varied widely and was shaped by governance arrangements and enabling environments, with missed opportunities for sustained coordination beyond the acute response phase [27,28]. Where roles are insufficiently integrated or mandates are unclear, preparedness systems may struggle to coordinate across sectors or adapt rapidly to evolving threats, even when individual technical capacities are present.

### Gender representation among identified stakeholder roles

Gender distribution represents another important dimension of preparedness capacity. Our findings show that 31.48% of identified stakeholder roles were associated with women, compared with 68.52% associated with men (Table 1). This pattern can be considered alongside global findings showing that women are widely represented in the health workforce but remain underrepresented in leadership and decision-making roles [29]. Similar disparities were observed during the COVID-19 response, with a global analysis of national task forces finding that most were predominantly male and that men held the majority of identified leadership positions [30]. Table 3 further illustrates that female representation varied across income groups and was highest in HICs (39.65%). Records associated with men predominated across all income groups, particularly in low-income countries (78.19% male) and upper-middle-income countries (76.94% male). These patterns highlight persistent gender imbalances in preparedness-related roles and raise concerns regarding the diversity of perspectives represented in preparedness decision-making. Greater gender inclusion in health emergency governance and leadership may contribute to more representative preparedness processes [30].

### Strengths and limitations

Methodologically, this study highlights both the strengths and limitations of stakeholder mapping based on publicly available sources. By drawing on organizational websites, reports, events, and professional profiles, the mapping captures actors who are visible within preparedness landscapes. Visibility is itself a relevant dimension of preparedness, as coordination and rapid mobilization depend on identifiable actors and accessible ways of coordination. Public visibility of stakeholders may facilitate communication, partnership formation, and cross-border collaboration during health emergencies. In parallel, differences in online presence, language, documentation practices, and politics across countries may influence the availability of this data. This introduces a visibility bias, whereby countries with weaker digital infrastructure, less systematic online documentation, or a lower likelihood for professionals and institutions to maintain digital profiles may be underrepresented. Stakeholders with a stronger digital presence are therefore more likely to be identified through this approach. To reduce this bias, the mapping drew on multiple source types, including official institutional websites, organizational reports, conference and webinar materials, and professional profiles. Searches were also conducted in both English and Arabic, and adapted to country-specific terminology when applicable. This may particularly affect conflict-affected settings such as Syria and Yemen, where disruptions to institutions and online documentation may reduce the visibility of preparedness actors. Nevertheless, preparedness assessments increasingly recognize transparency, traceability, and communication as integral to system readiness [31], suggesting that gaps in visibility may also signal governance and coordination challenges that can present difficulties in times of crises. Consequently, the mapping reflects visible preparedness ecosystems rather than the full underlying workforce. Classification of stakeholder roles into preparedness domains and workforce categories was also based on publicly described roles and may oversimplify complex or multi-functional positions. Finally, this study does not assess the effectiveness, performance, or influence of stakeholders within preparedness systems, and findings should therefore be interpreted as descriptive of the distribution of identified preparedness-related roles and affiliations rather than direct measures of preparedness capacity.

## Conclusions

The findings of this study point to the need for preparedness in the MENA region to focus not only on expanding capacity but considering how core preparedness roles are distributed across different functions and embedded within the existing health systems. While preparedness activity is present across the region, the greater representation of identified roles within operational domains may indicate a stronger emphasis on operational functions compared with strategic functions that support planning, policy development, and sustained communication capacity beyond periods of crisis response. The comparatively lower representation of identified roles in emergency risk communication and policy development warrants further assessment to understand whether this reflects differences in workforce composition, institutional priorities, stakeholder visibility, or other country-level factors. Strengthening country-level preparedness and promoting greater gender representation may enhance inclusivity, and long-term resilience across the region. Stakeholder mapping can play a practical role in this process by helping identify areas where representation is comparatively limited and where coordination and capacity-building efforts may be most needed. Stakeholder mapping provides a practical approach for improving the visibility of preparedness systems, particularly in regions where institutional roles and responsibilities are not clearly documented. Understanding these patterns can support more targeted discussions around how preparedness functions are organized and represented across different settings. Future work could strengthen the study’s approach by validating mapped roles and affiliations through engagement with key informants and institutions, examining stakeholder interests and influence, and assessing communication, engagement, and relationships between actors within the preparedness landscape, and assessing how stakeholder distribution influences preparedness performance during outbreaks.

## Supporting information

S1 Table Keywords Used to Guide Stakeholder Identification and Mapping. (DOCX)

S2 Table Identified stakeholder-role records by country and population-standardized values. (DOCX)

S1 Fig Country-level Profiles Across MENA. (PDF)

S1 Data Stakeholder Mapping Dataset. (XLSX)

S1 Checklist STROBE Checklist. (DOC)
