# Supplementary material for: Epidemic and pandemic preparedness in the MENA region: A descriptive analysis of stakeholder distribution, patterns, and regional variation
Source: PLOS Glob Public Health. 2026 Nov 6;6(11):e0007574. doi: 10.1371/journal.pgph.0007574 (PMC13742938; doi:10.1371/journal.pgph.0007574)

ALGERIA

Population
47.435 M

Income Level
Upper Middle

GHS Index Score
26.2

Individual Stakeholders 187

68% Males

32% Females

Stakeholders Organizations 85

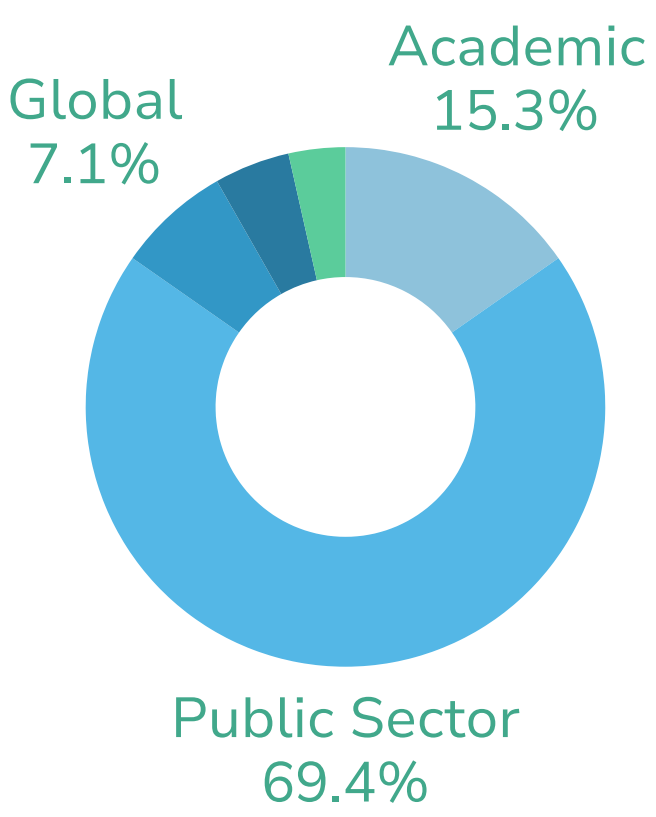

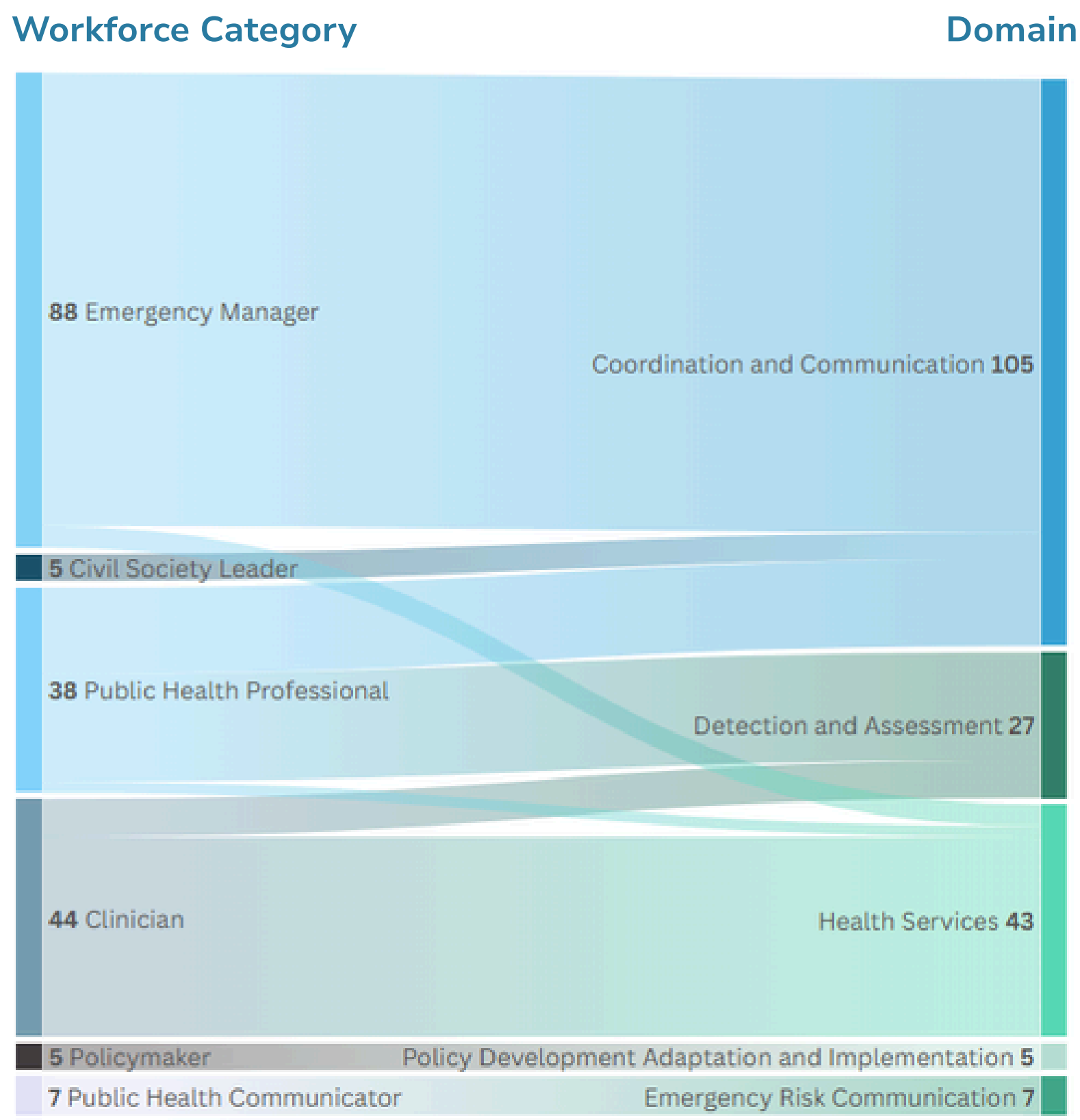

BAHRAIN

Population

1.643M

Income Level

High

GHS Index Score

36.3

Individual Stakeholders 193

51% Males

49% Females

Stakeholders Organizations 61

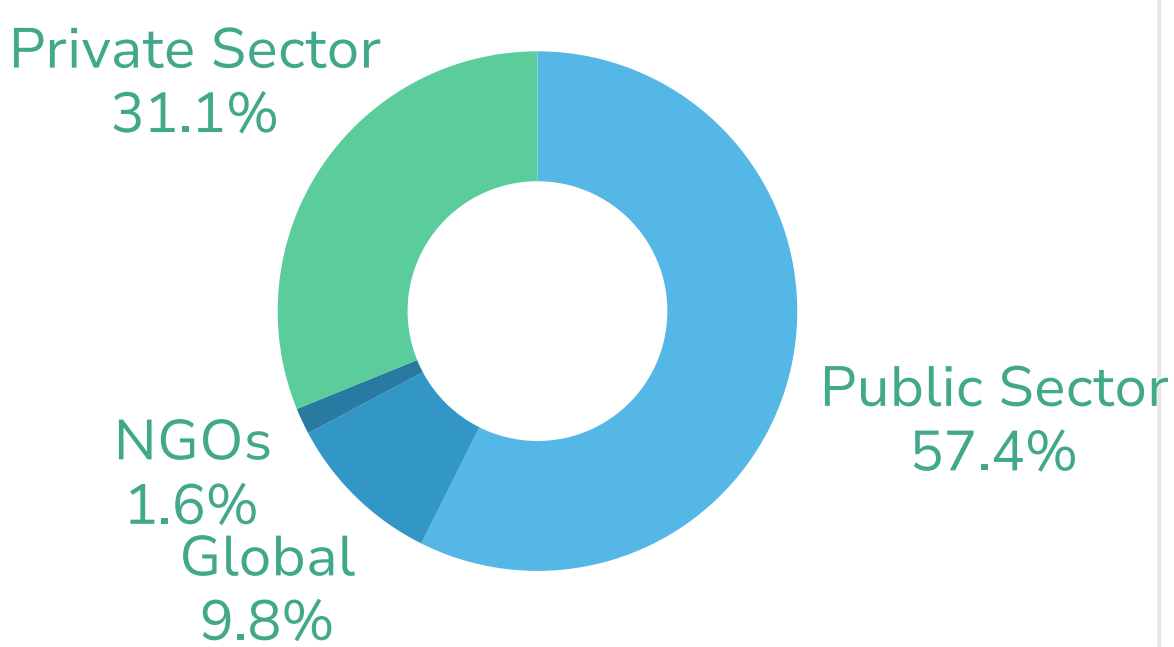

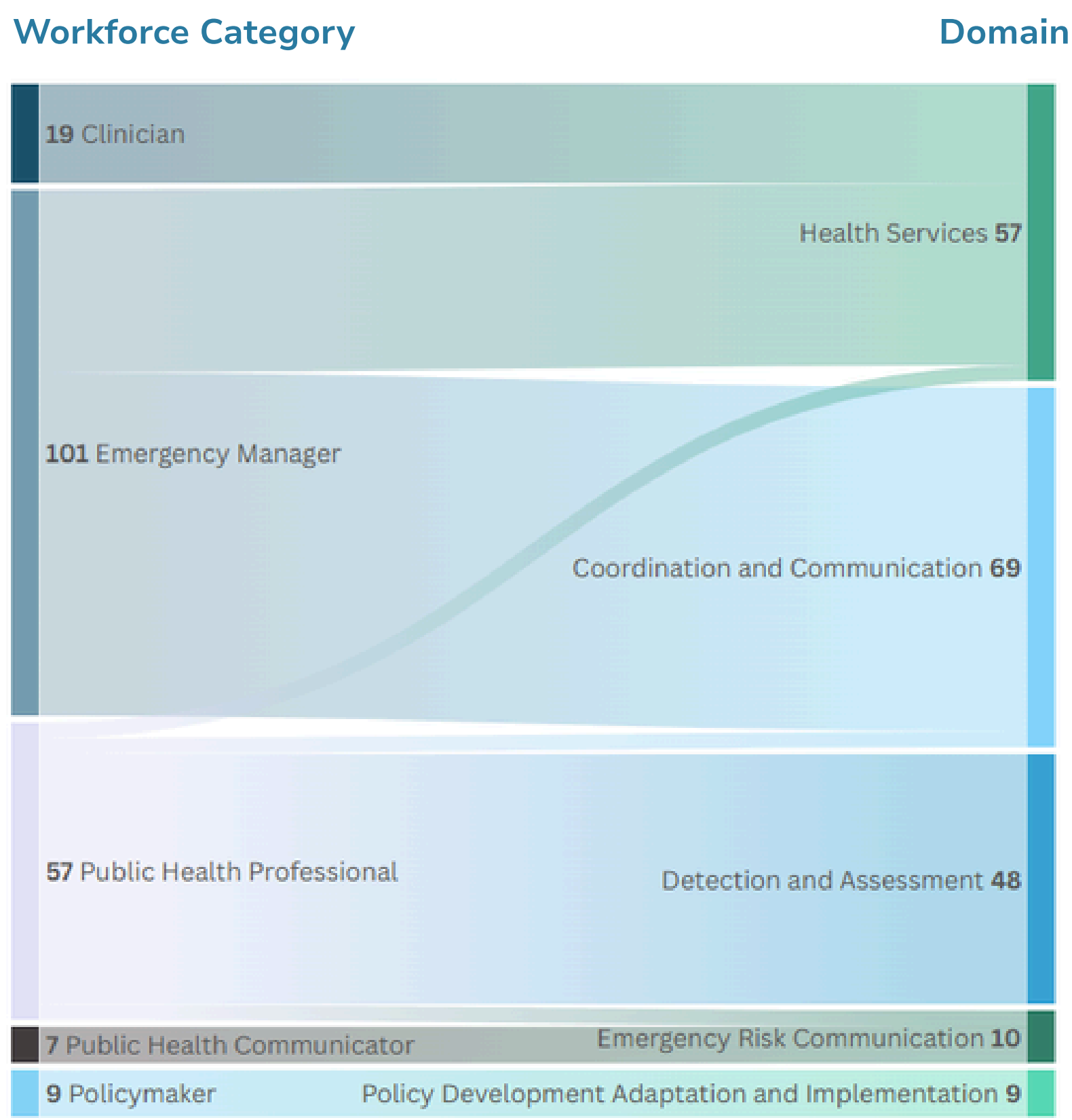

COMOROS

Population
0.882 M

Income Level
Low Middle

GHS Index Score
24.9

Individual Stakeholders 51

73% Males
27% Females

Stakeholders Organizations 17

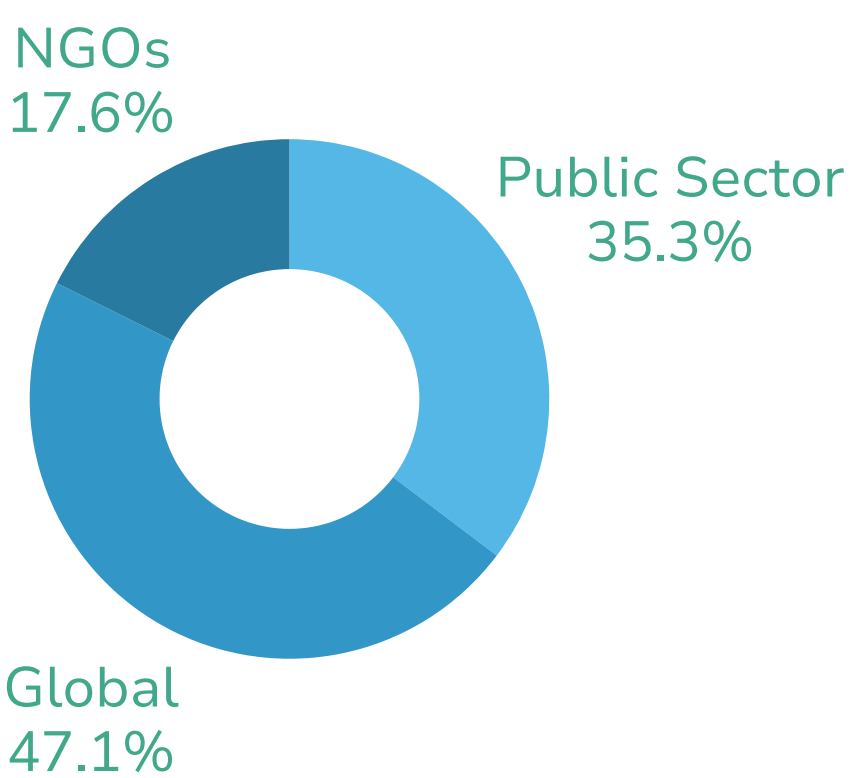

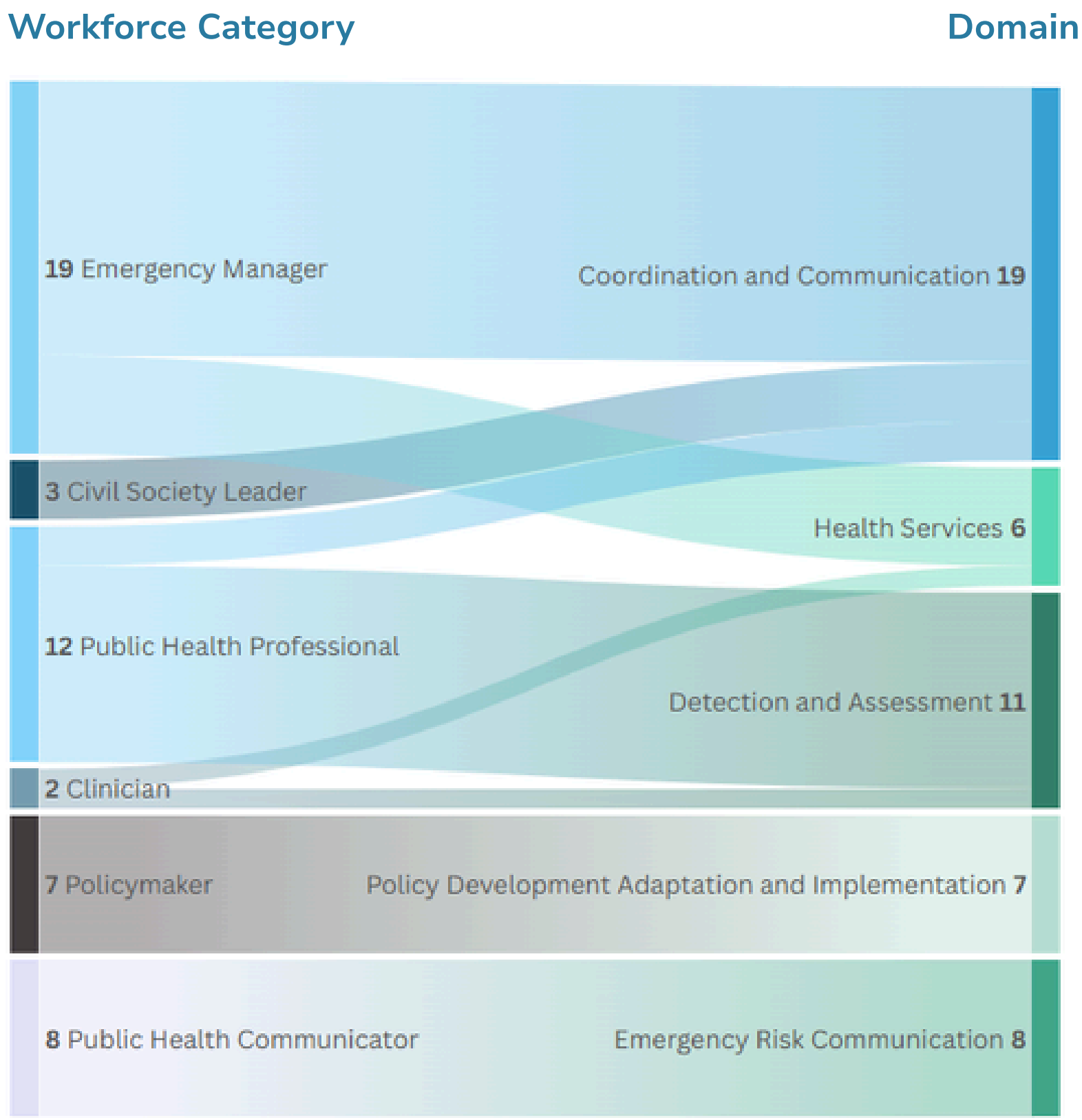

DJIBOUTI

Population
1.184 M

Income Level
Low Middle

GHS Index Score
25.2

Individual Stakeholders 52

67% Males
33% Females

Stakeholders Organizations 15

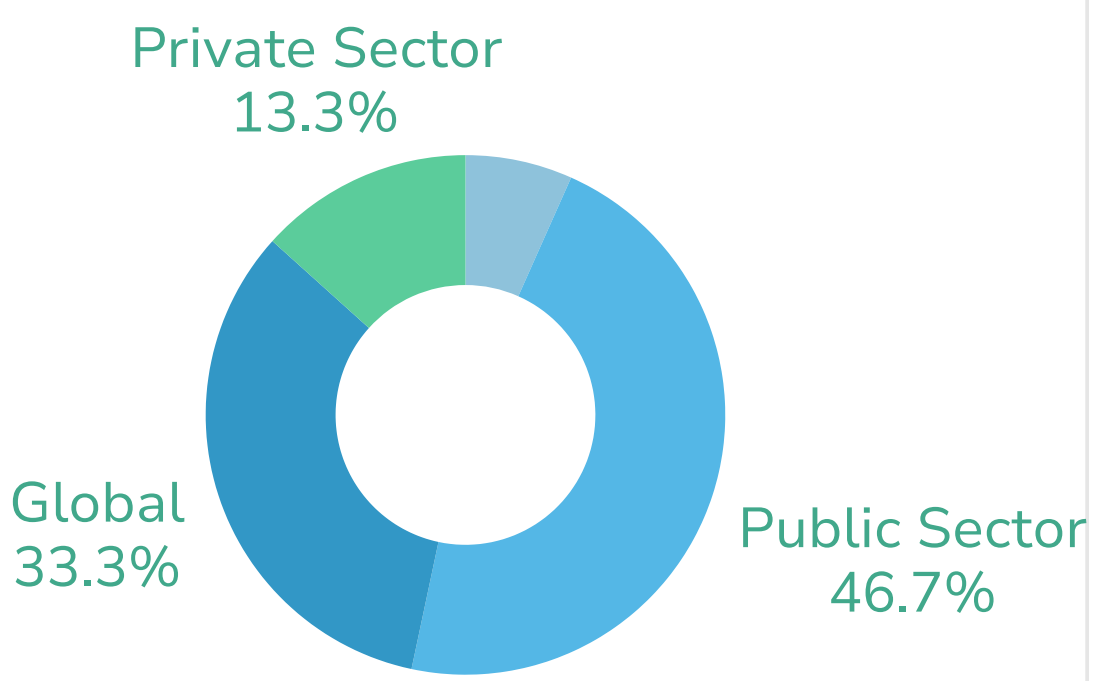

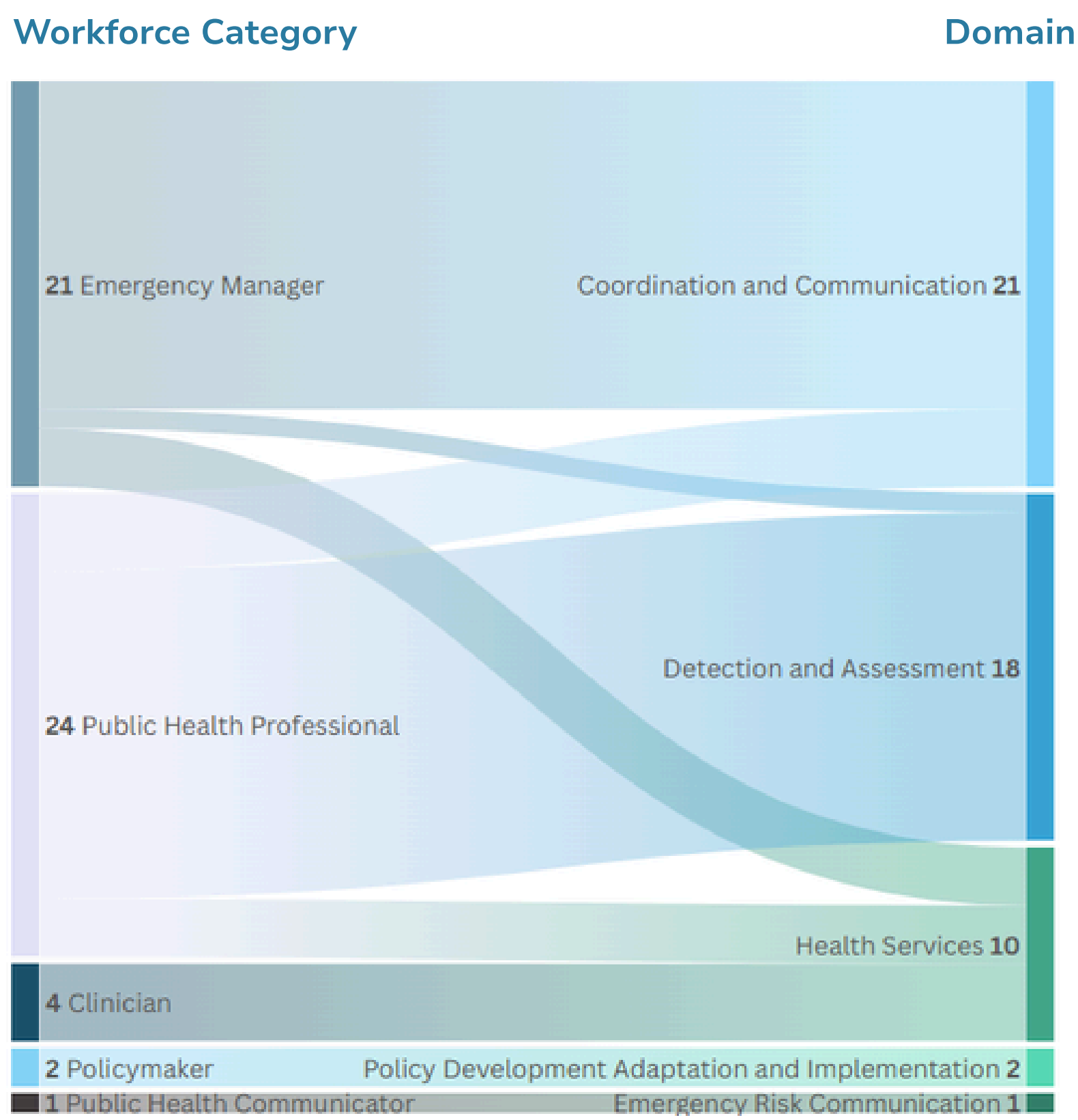

EGYPT

Population
118.365 M

Income Level
Low Middle

GHS Index Score
28

Individual Stakeholders 324

60% Males
40% Females

Stakeholders Organizations 66

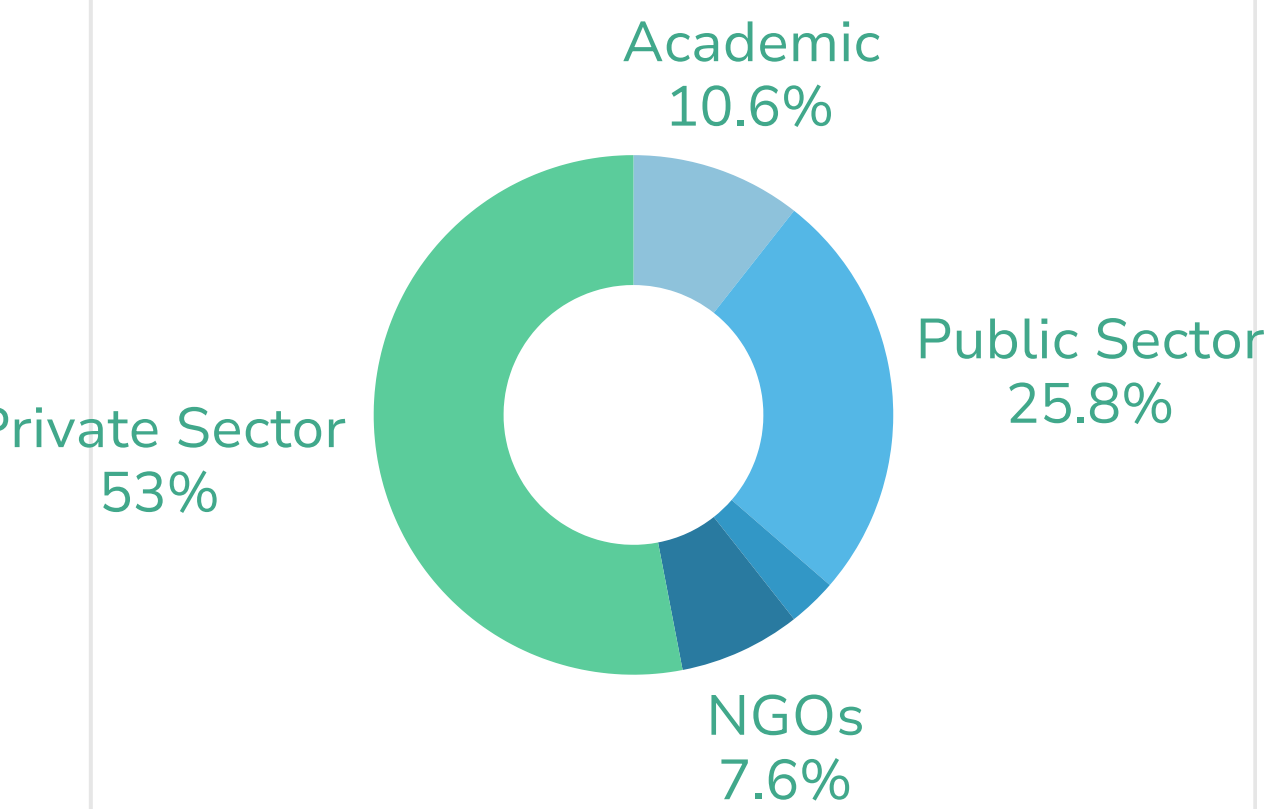

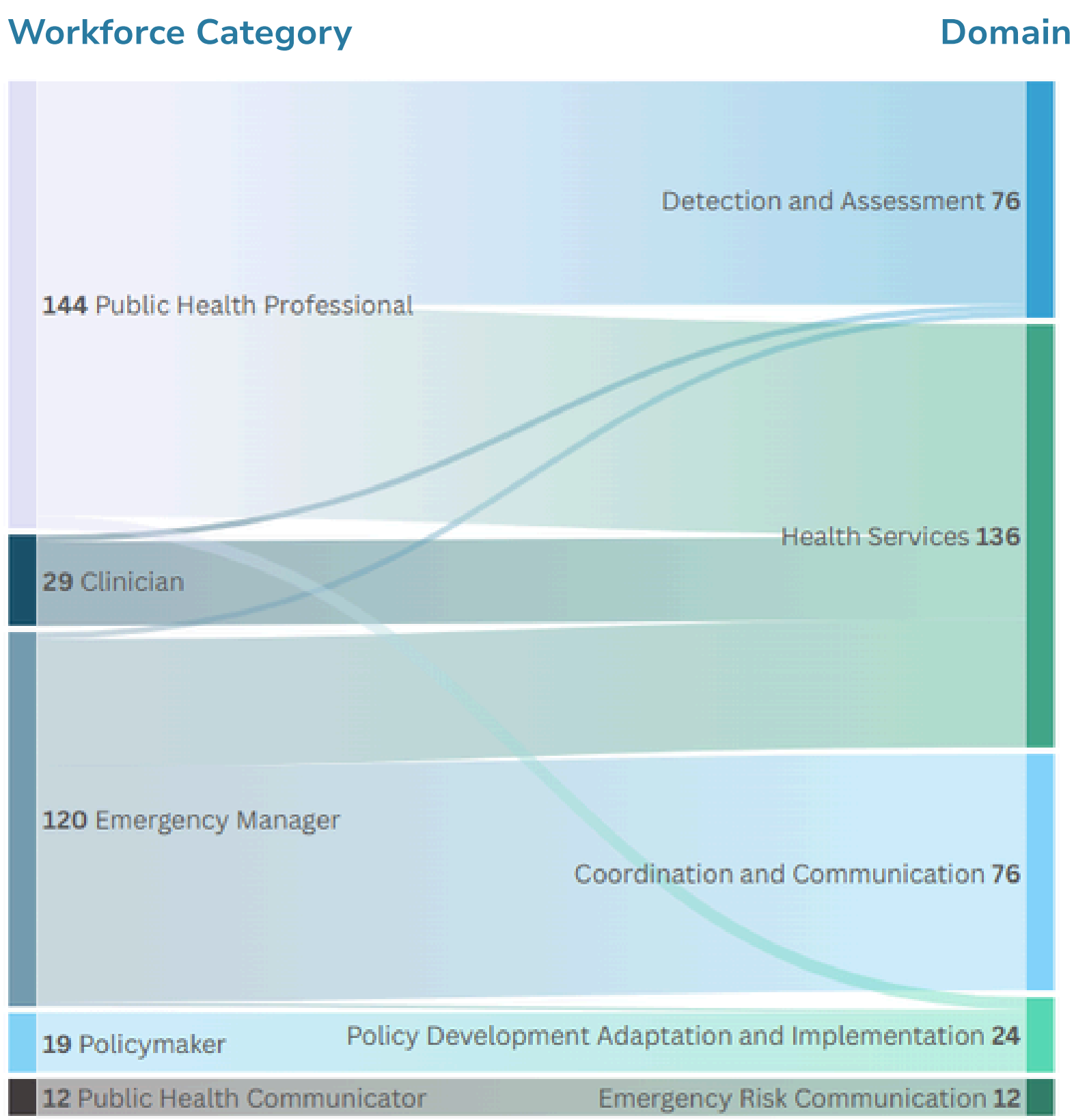

IRAQ

Population
47.020 M

Income Level
Upper Middle

GHS Index Score
24

Individual Stakeholders 97

78% Males   22% Females

Stakeholders Organizations 26

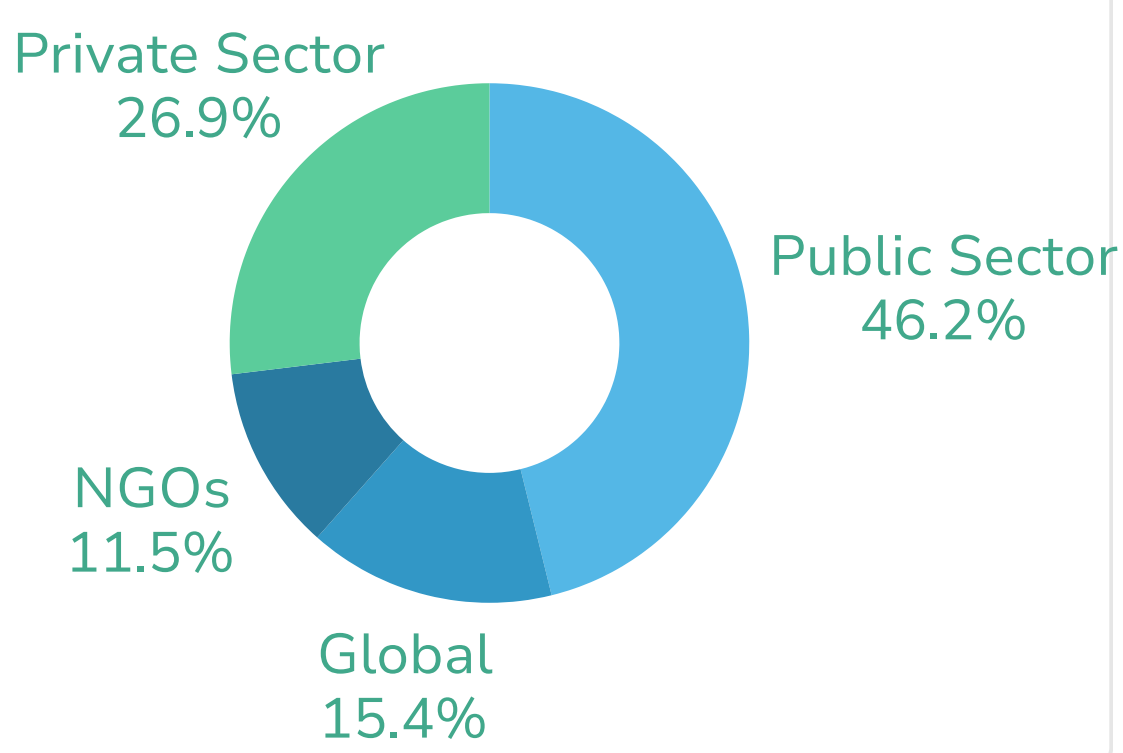

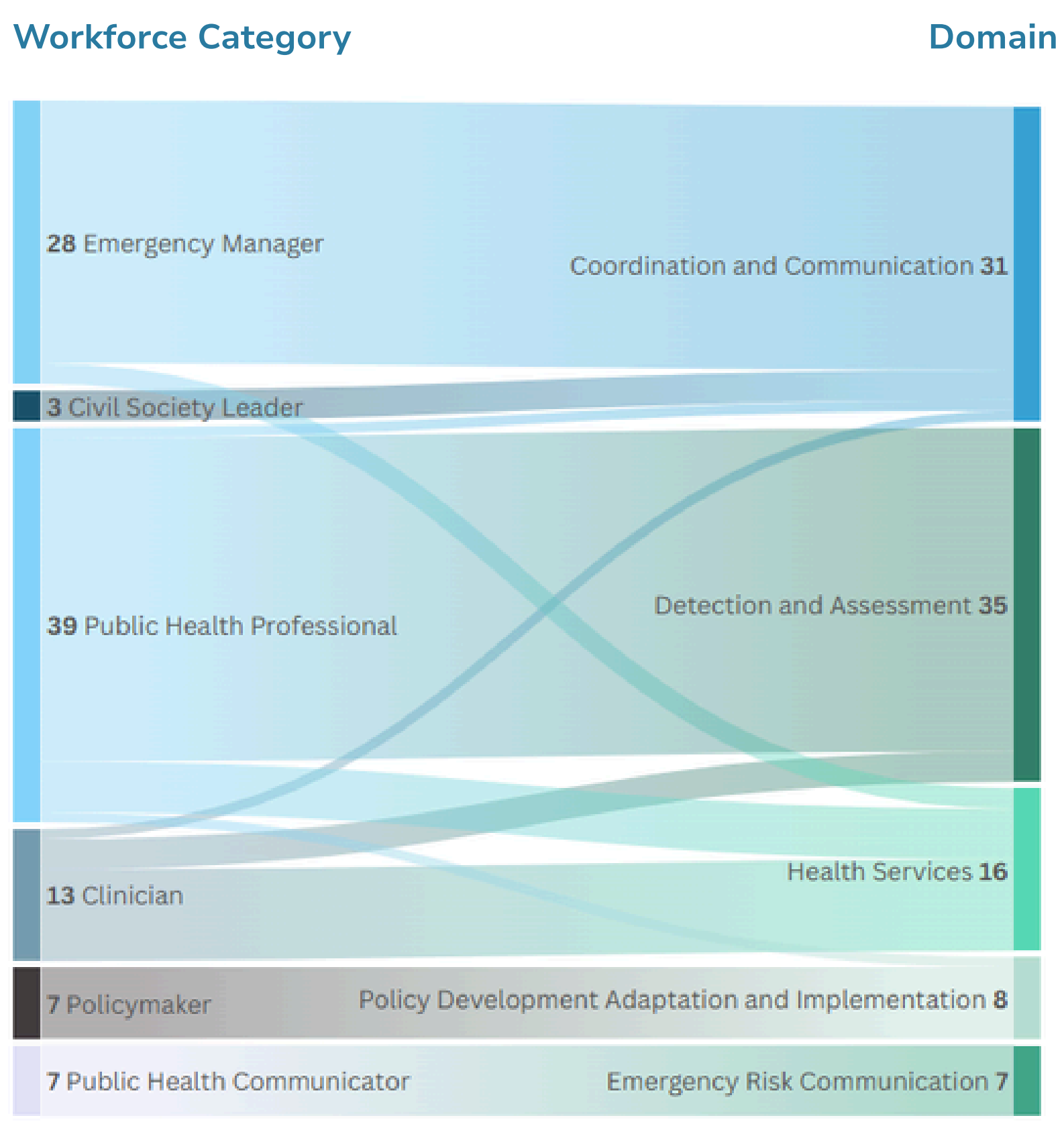

JORDAN

Population
11.520 M

Income Level
Low Middle

GHS Index Score
42.8

Individual Stakeholders 147

71% Males

29% Females

Stakeholders Organizations 54

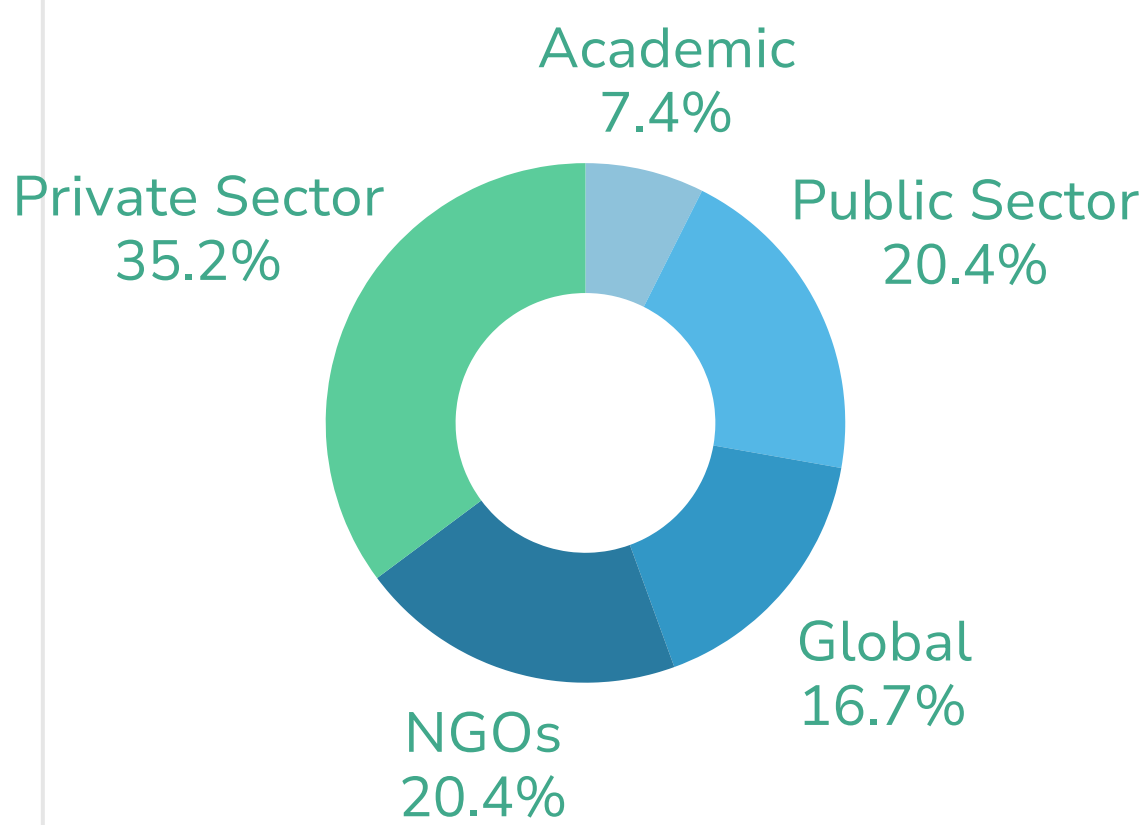

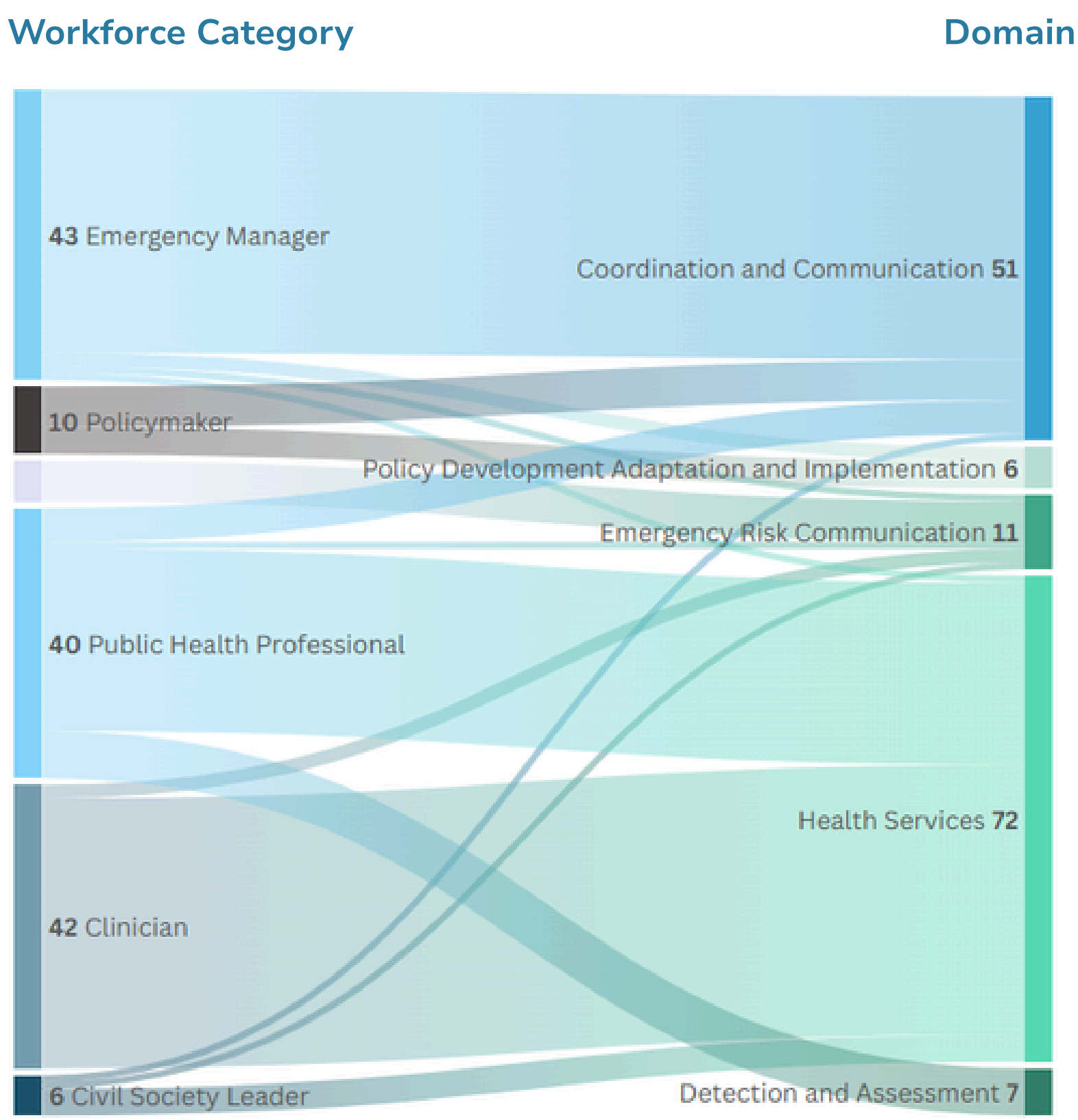

KUWAIT

Population
5.026 M

Income Level
High

GHS Index Score
36.8

Individual Stakeholders 130

60% Males
40% Females

Stakeholders Organizations 28

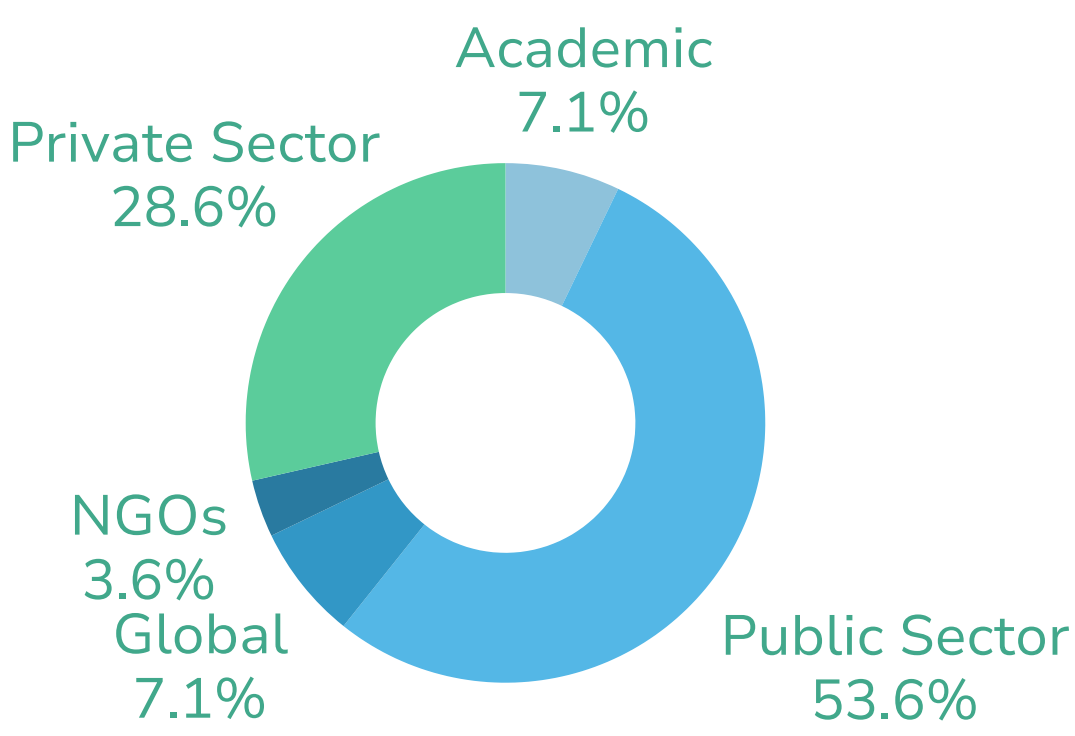

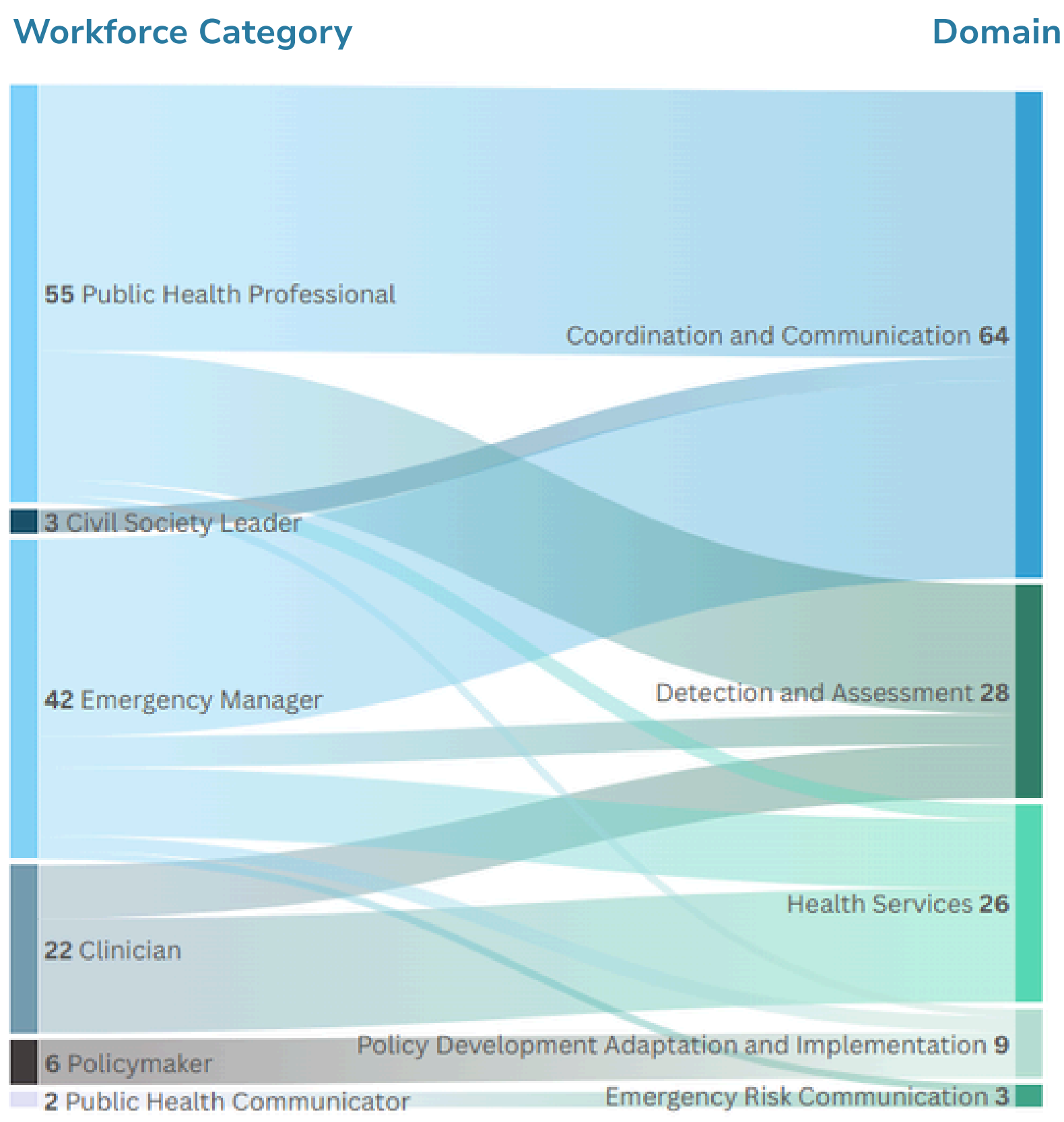

LEBANON

Population
5.849 M

Income Level
Low Middle

GHS Index Score
33.4

Individual Stakeholders 341

72% Males

28% Females

Stakeholders Organizations 103

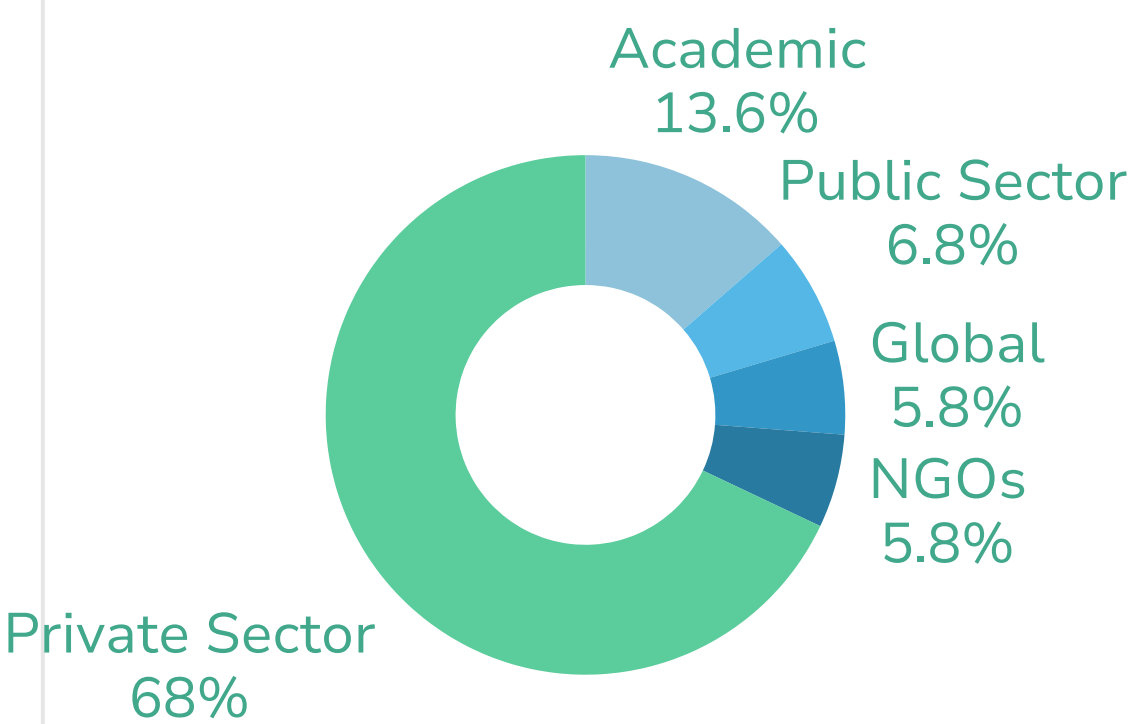

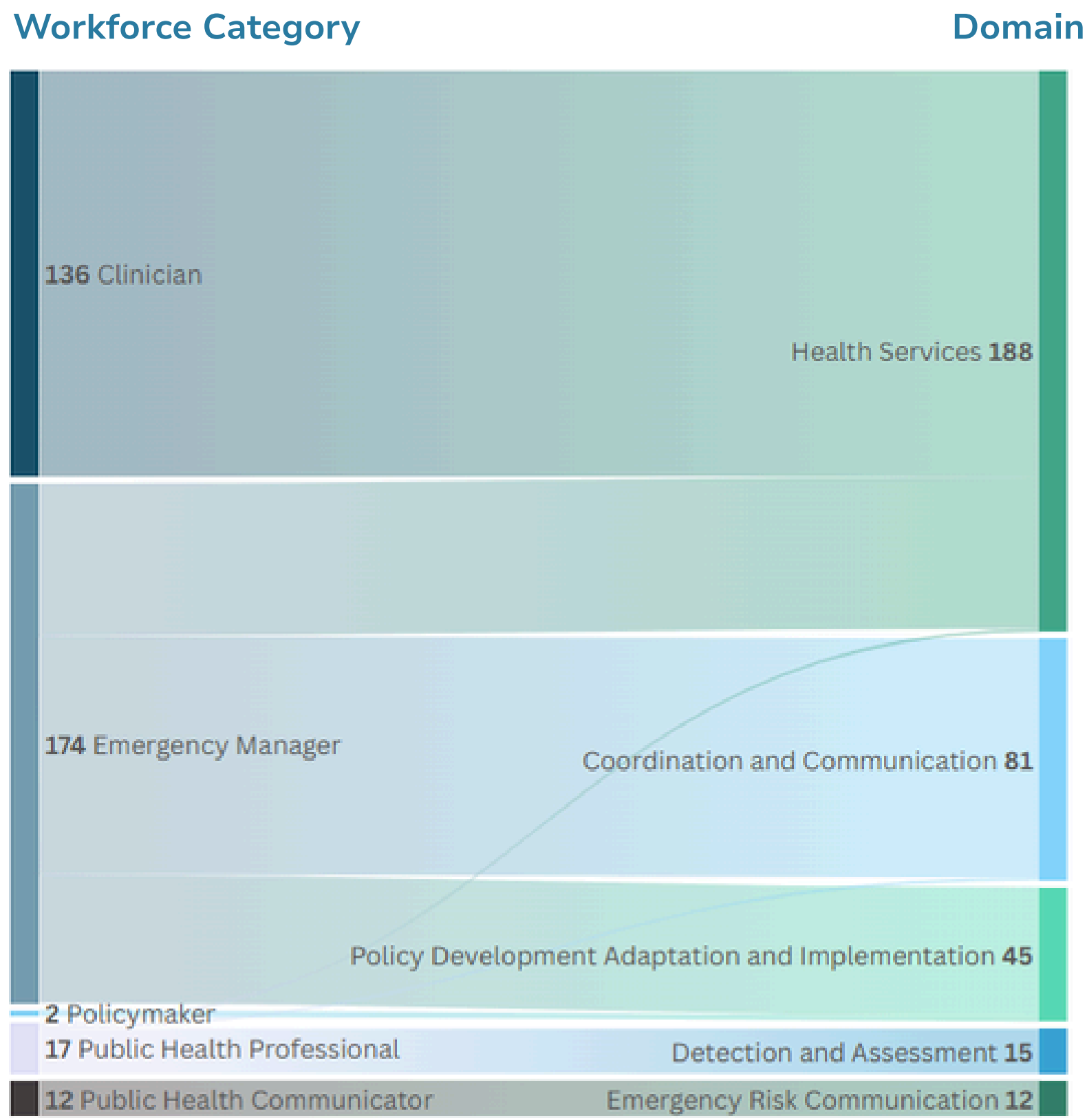

LIBYA

Population
7.458 M

Income Level
Upper Middle

GHS Index Score
25.3

Individual Stakeholders 128

88% Males

12% Females

Stakeholders Organizations 23

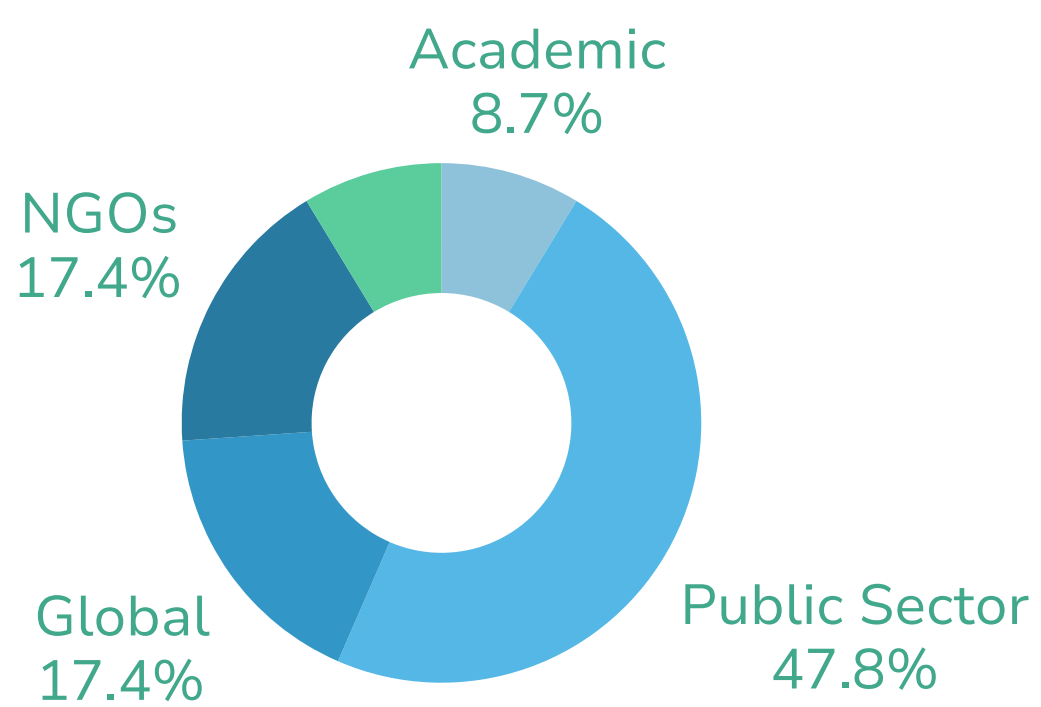

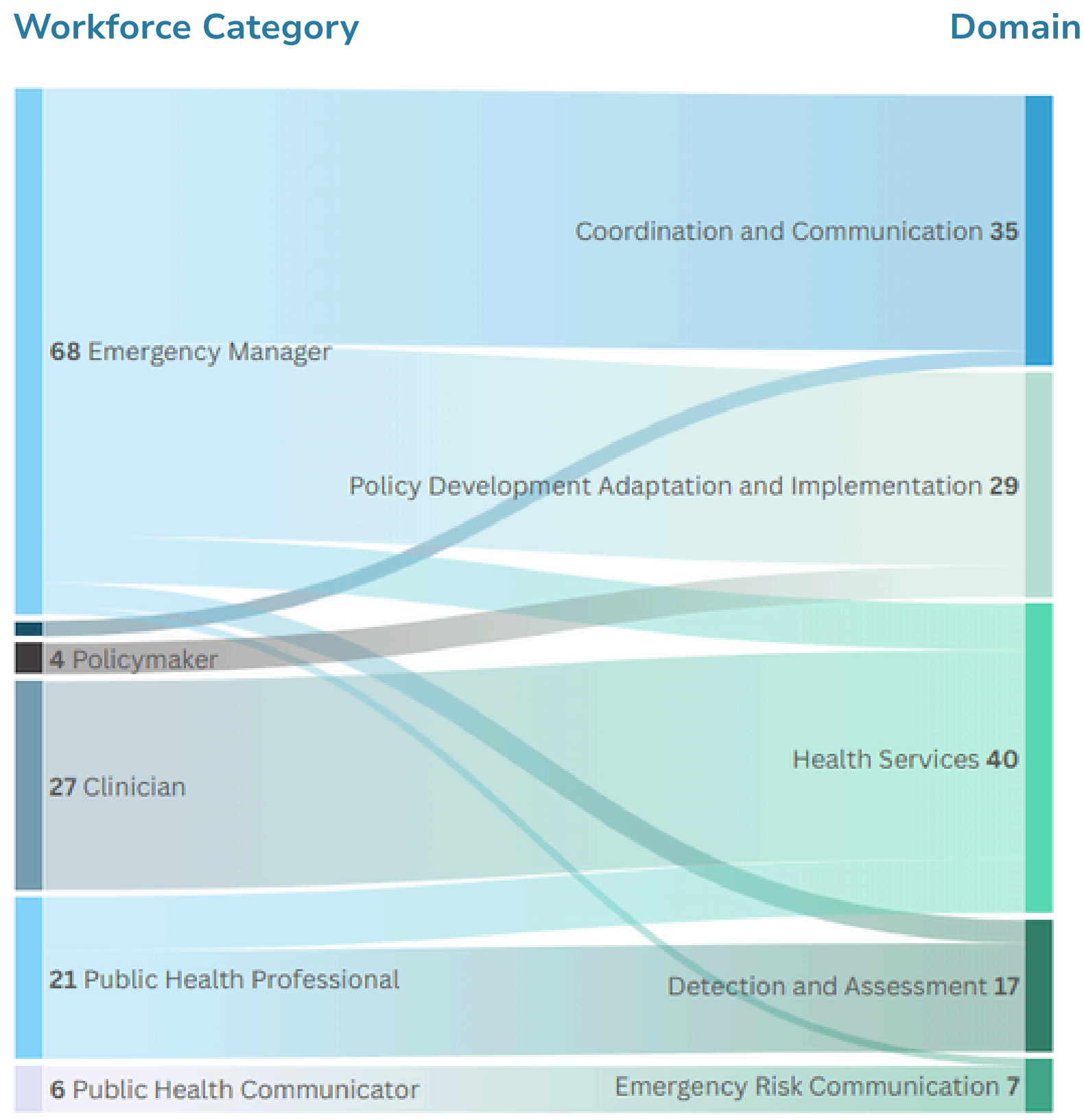

MAURITANIA

Population
5.315 M

Income Level
Low Middle

GHS Index Score
26.2

Individual Stakeholders 42

74% Males

26% Females

Stakeholders Organizations 13

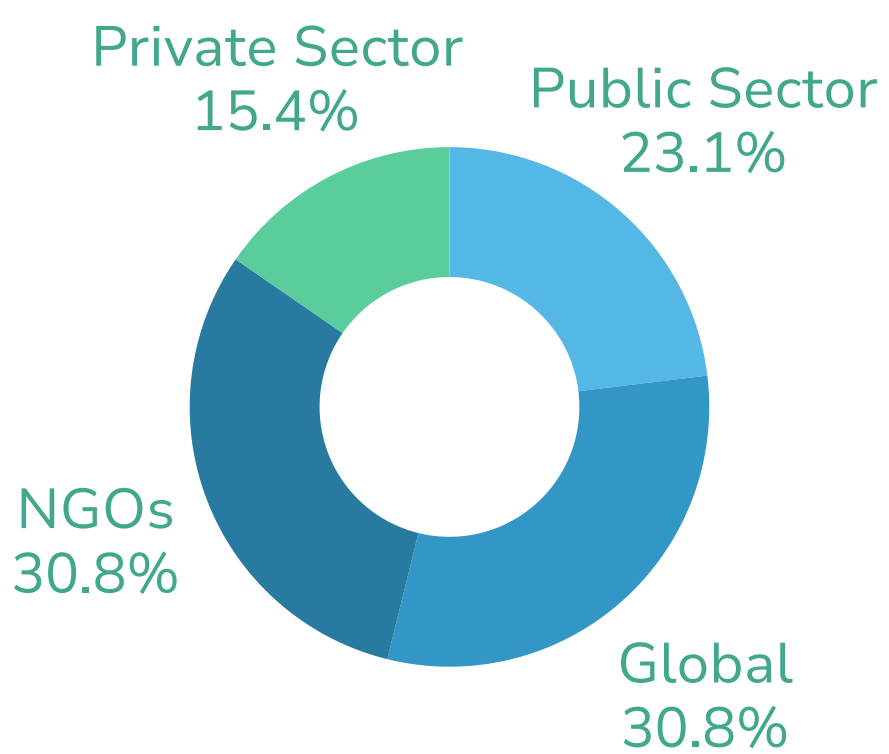

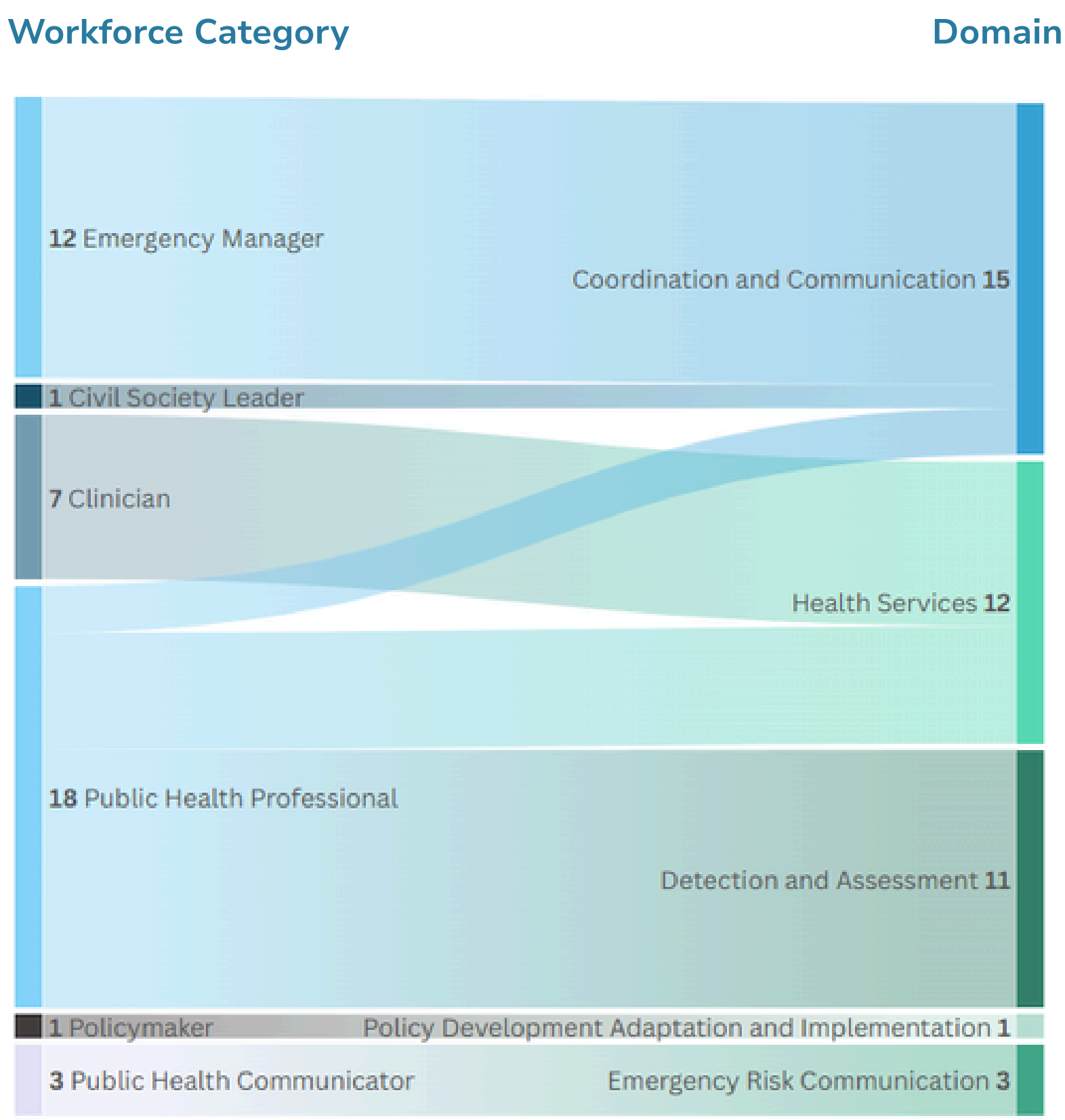

MOROCCO

Population
38.430 M

Income Level
Low Middle

GHS Index Score
33.6

Individual Stakeholders 160

64% Males

36% Females

Stakeholders Organizations 55

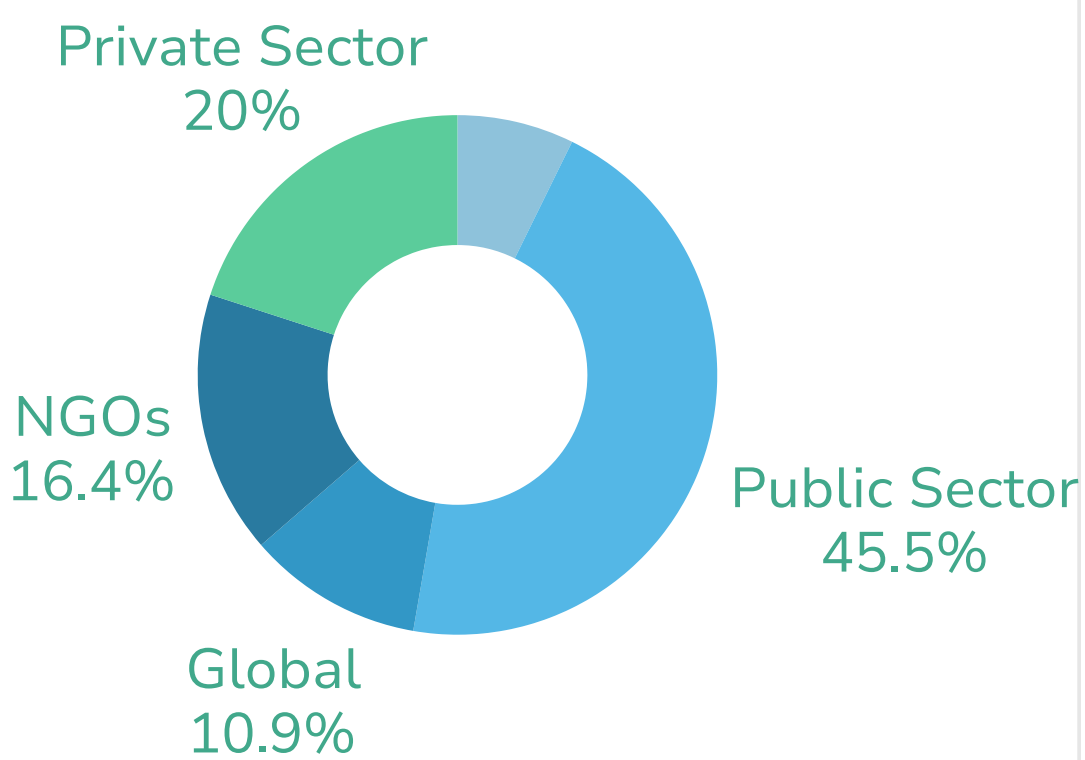

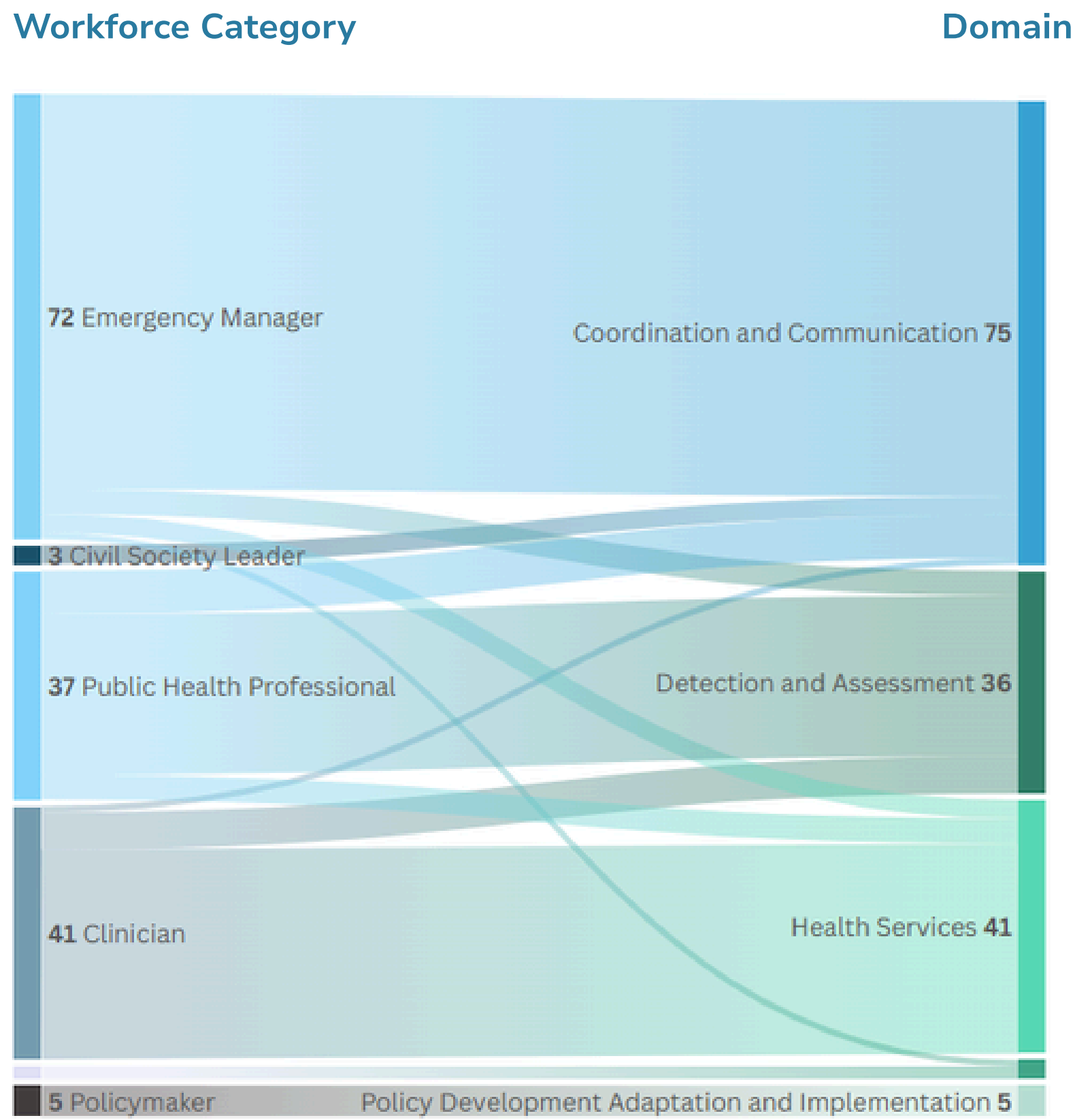

OMAN

Population
5.494 M

Income Level
High

GHS Index Score
39.1

Individual Stakeholders 140

69% Males
31% Females

Stakeholders Organizations 22

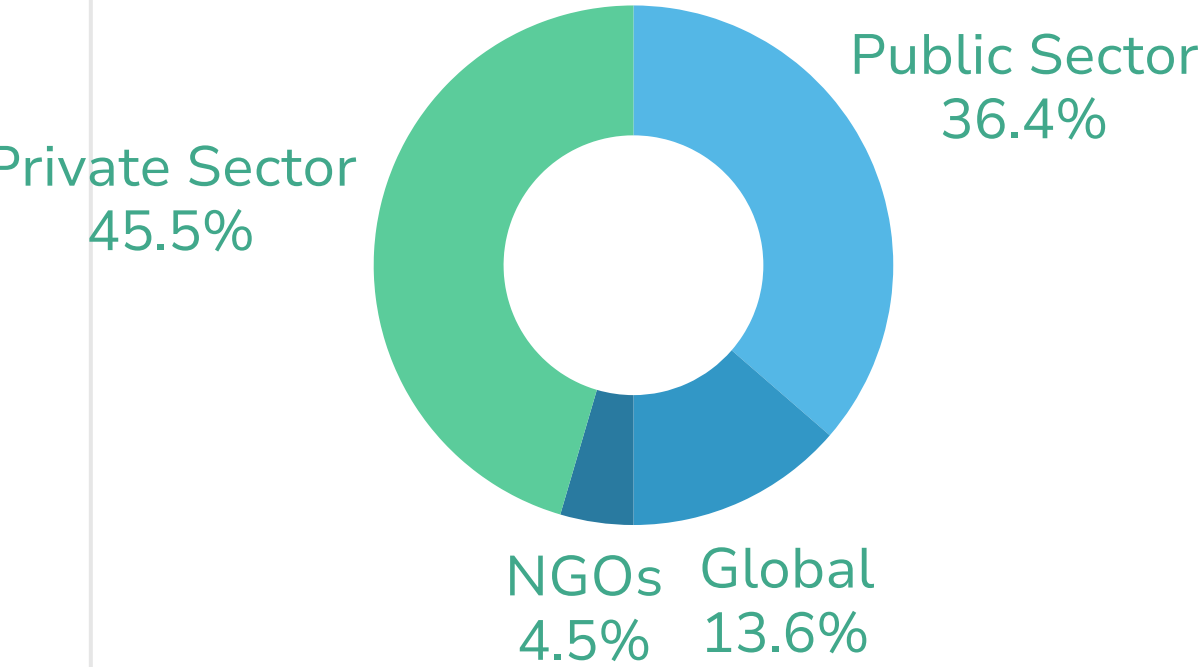

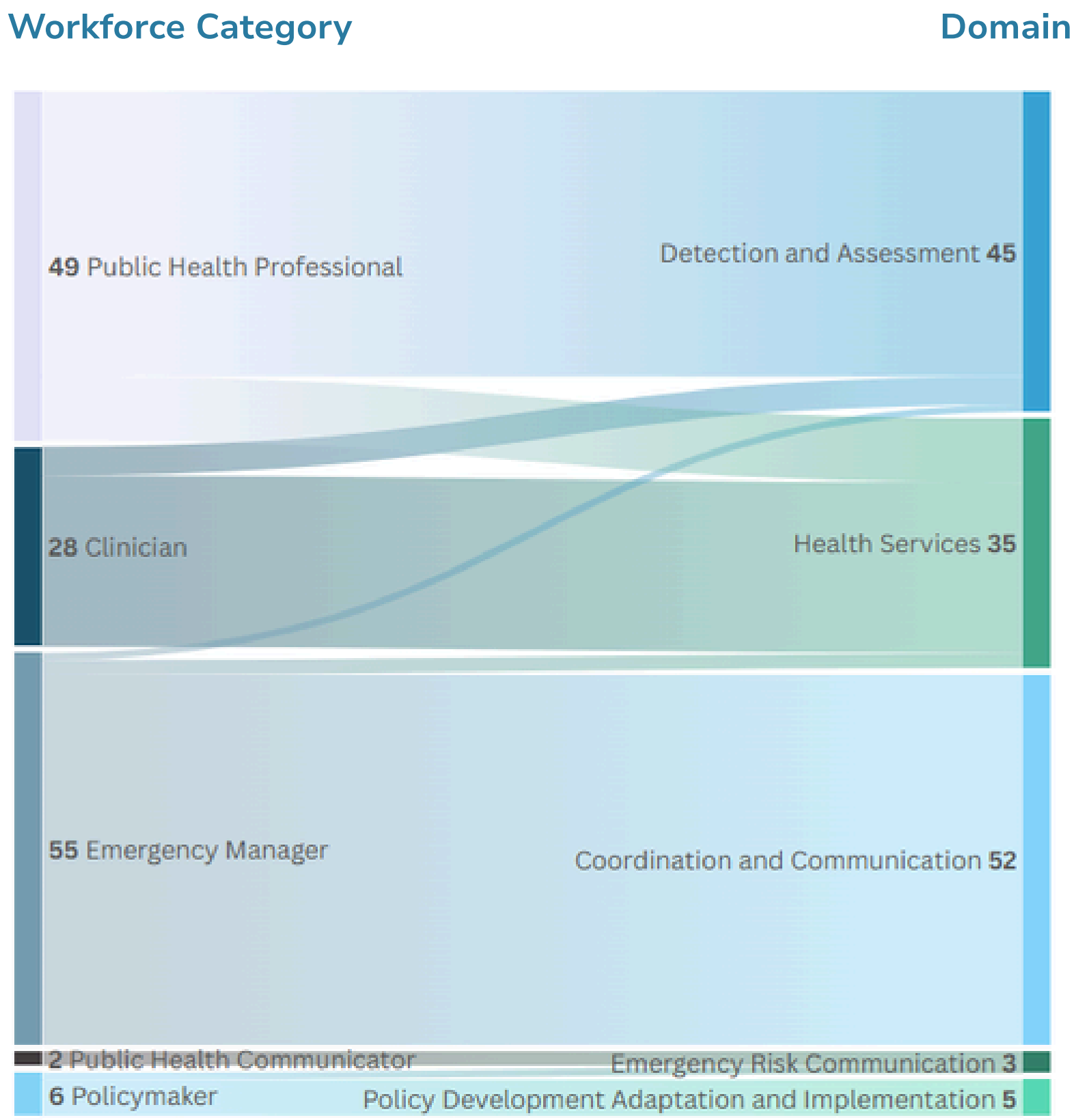

PALESTINE

Population
5.589 M

Income Level
Low Middle

GHS Index Score
Not Applicable

Individual Stakeholders 326

82% Males

18% Females

Stakeholders Organizations 84

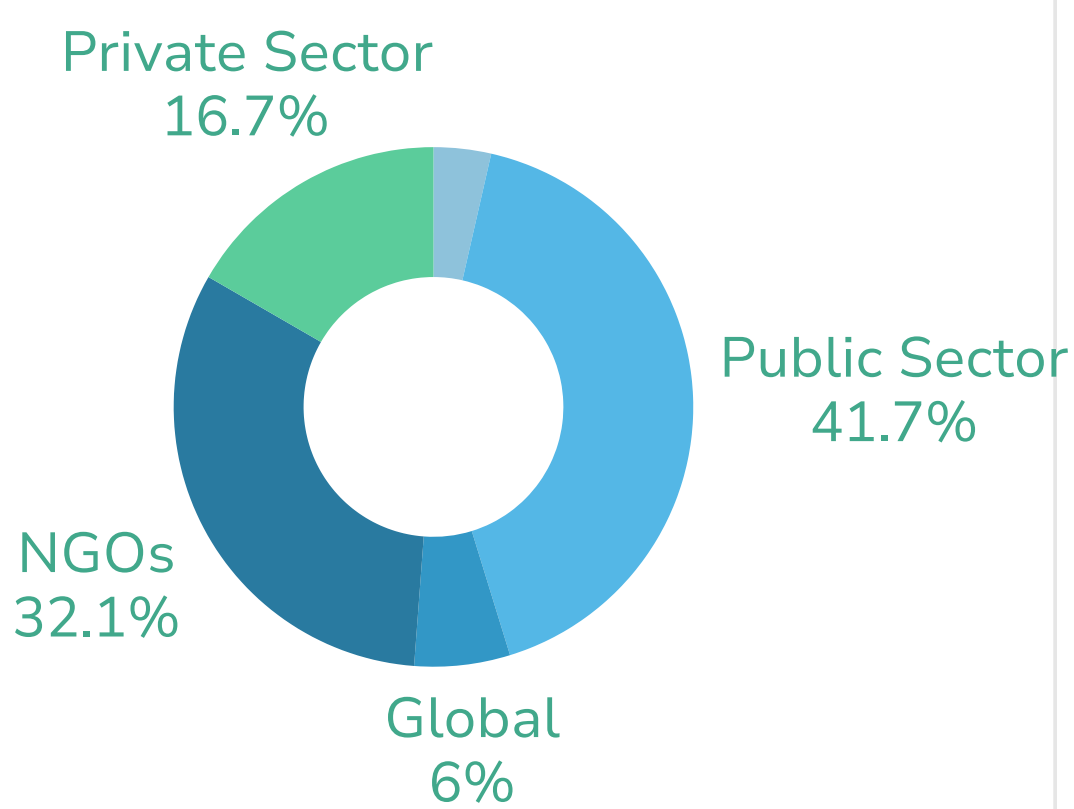

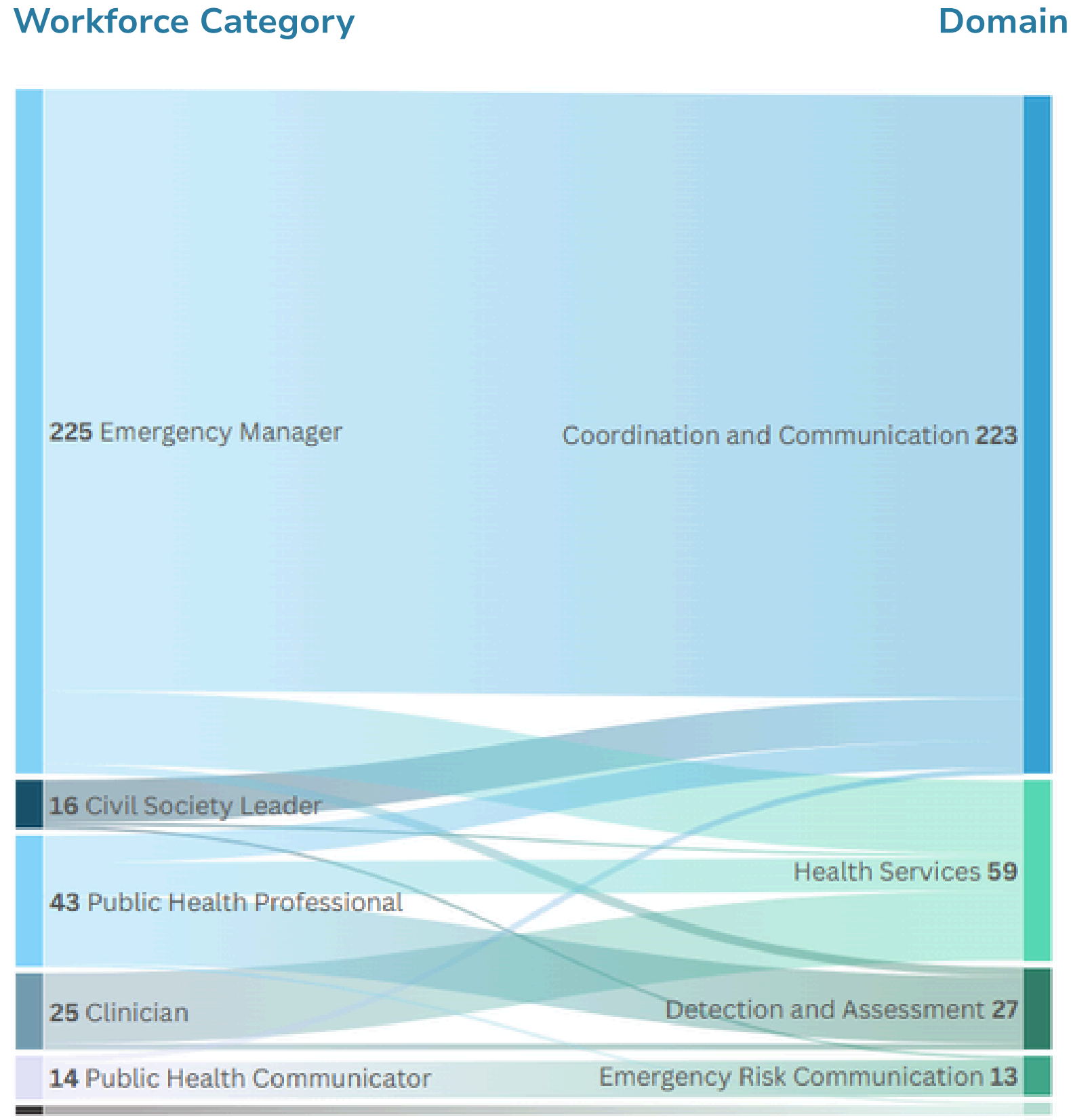

QATAR

Population
3.115 M

Income Level
High

GHS Index Score
48.7

Individual Stakeholders 102

72% Males

28% Females

Stakeholders Organizations 10

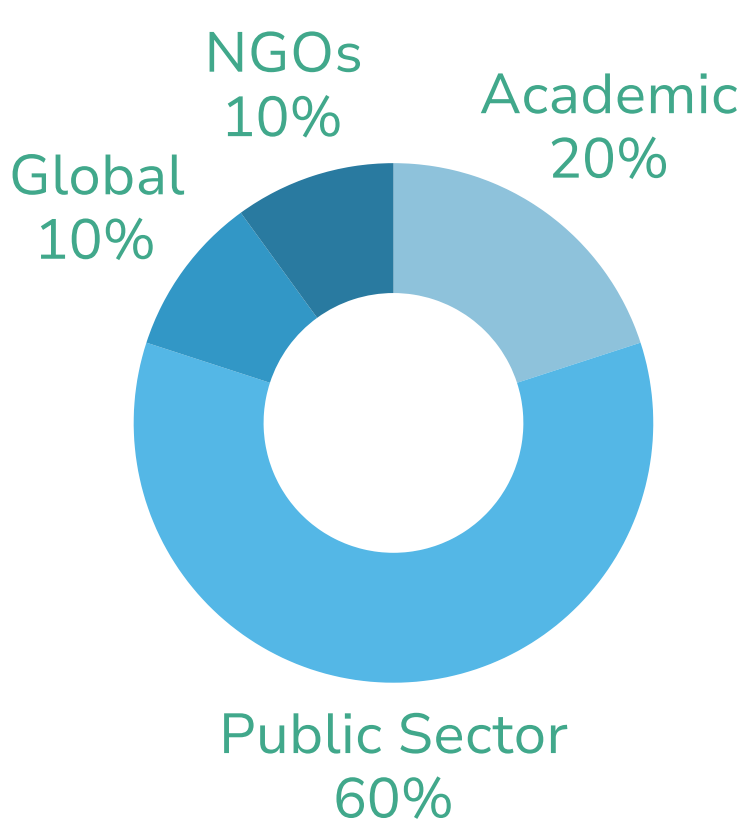

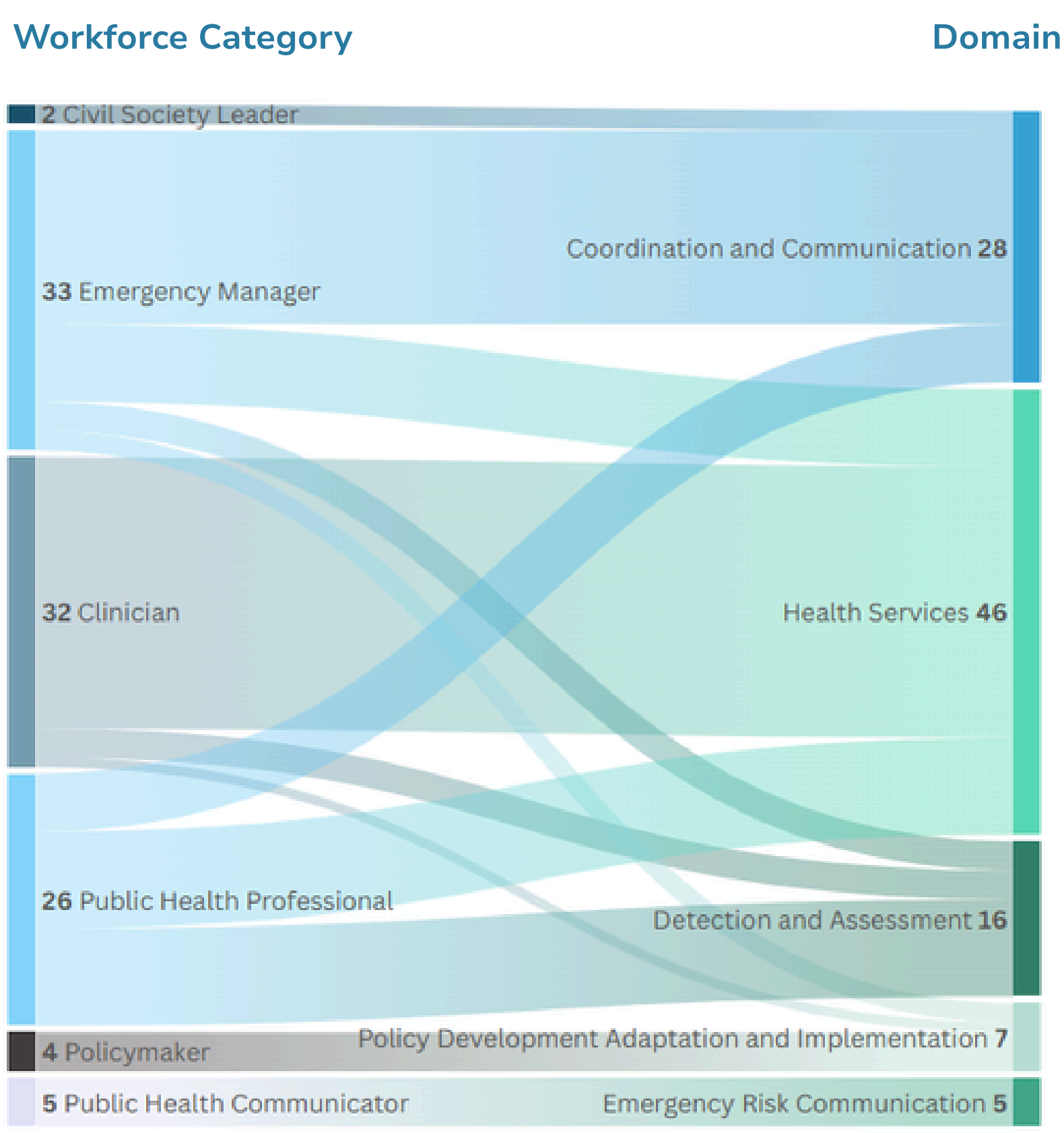

SAUDI ARABIA

Population
34.566 M

Income Level
High

GHS Index Score
44.9

Individual Stakeholders 94

74% Males

26% Females

Stakeholders Organizations 27

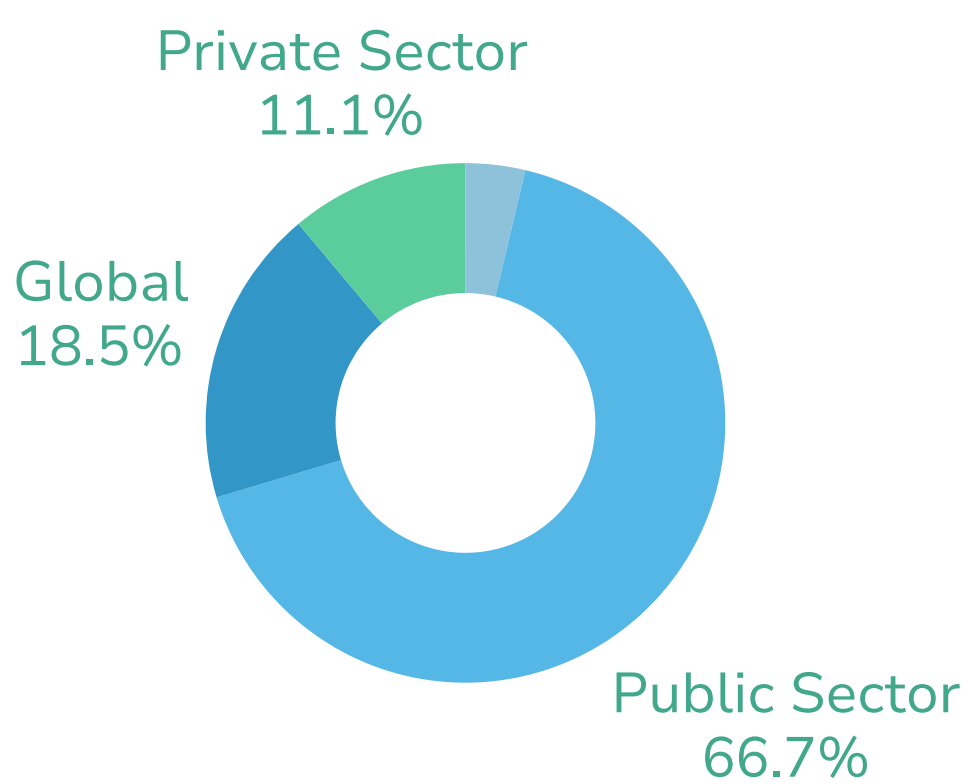

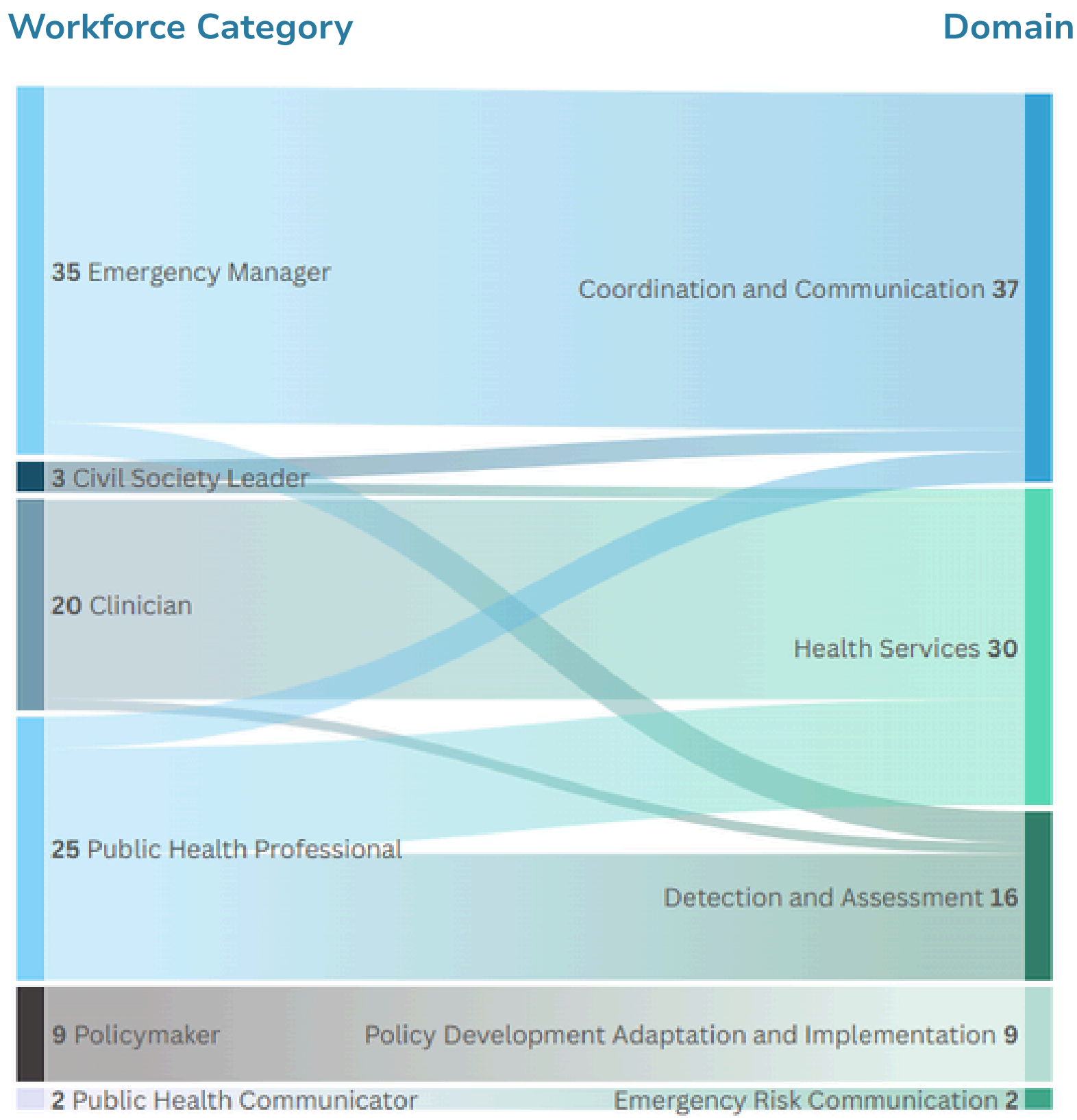

SOMALIA

Population
19.654 M

Income Level
Low

GHS Index Score
16

Individual Stakeholders 177

89% Males

11% Females

Stakeholders Organizations 28

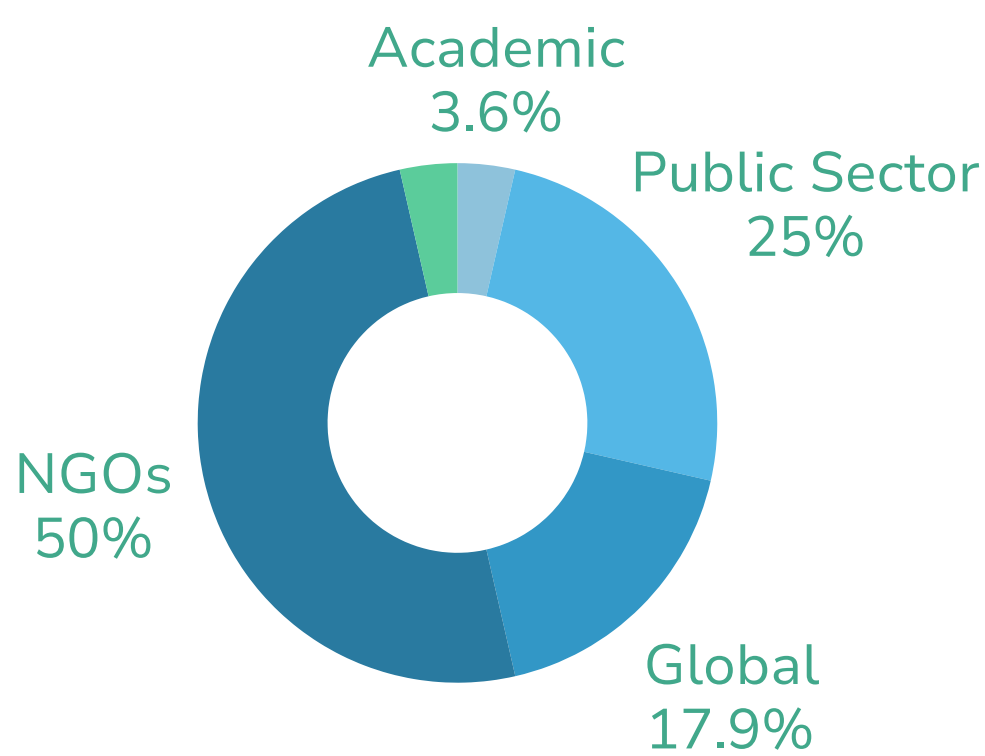

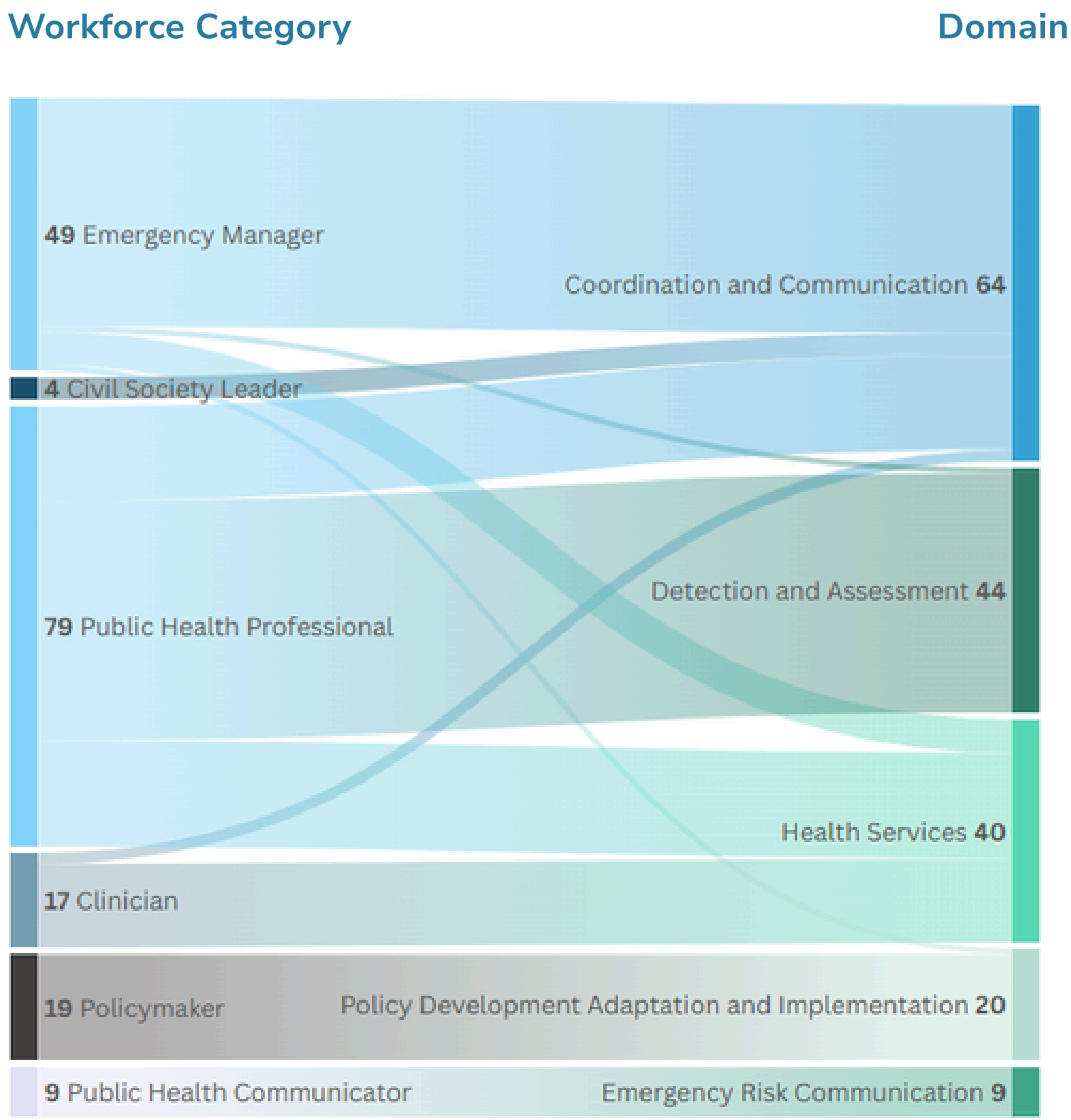

SUDAN

Population
51.662 M

Income Level
Low

GHS Index Score
28.3

Individual Stakeholders 170

67% Males
33% Females

Stakeholders Organizations 34

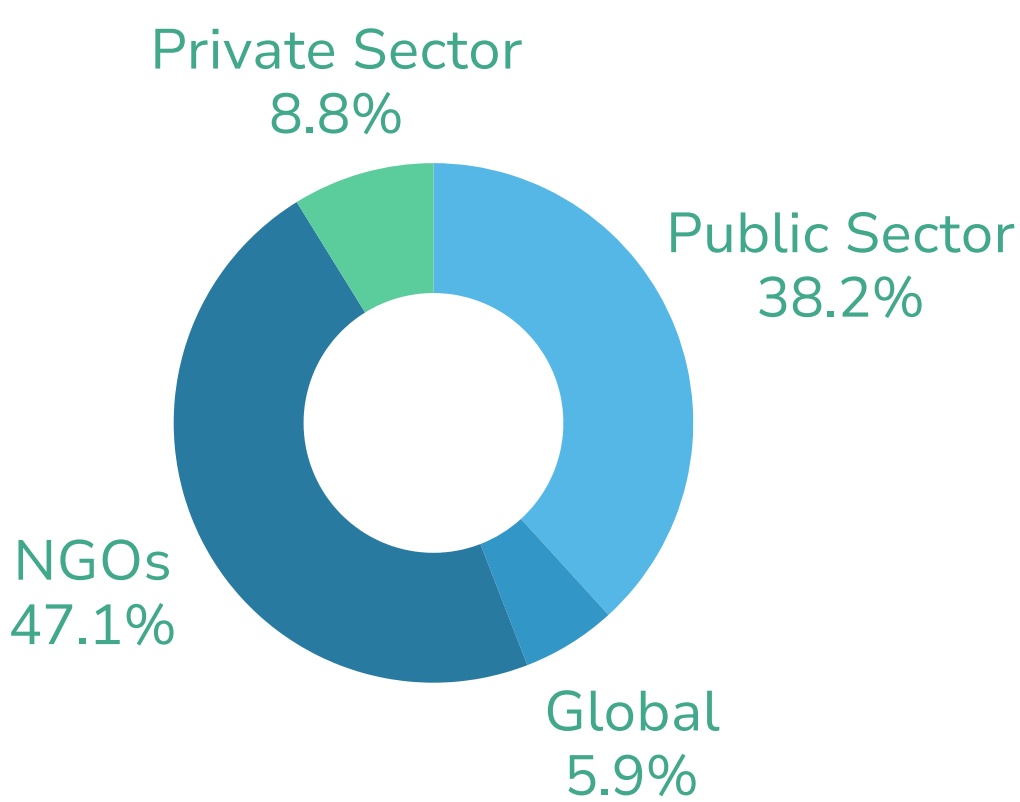

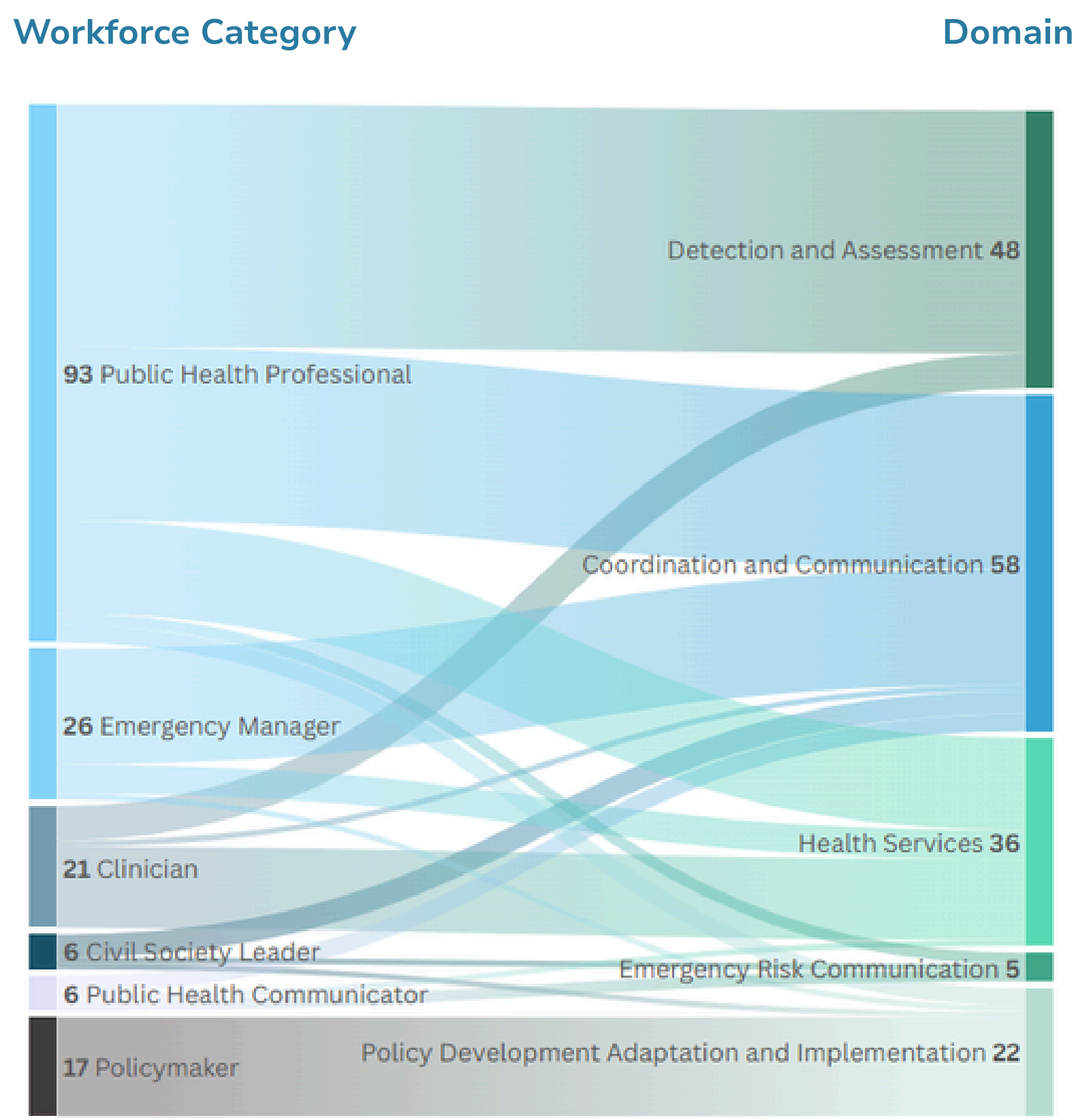

SYRIA

Population
25.620 M

Income Level
Low

GHS Index Score
16.7

Individual Stakeholders 42

62% Males   38% Females

Stakeholders Organizations 24

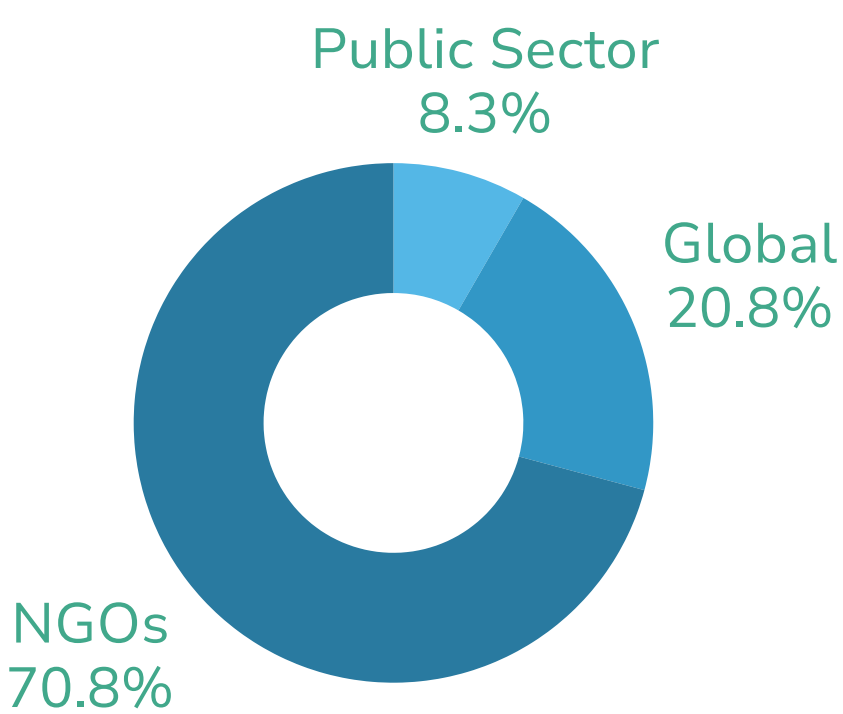

Workforce Category | **Domain**

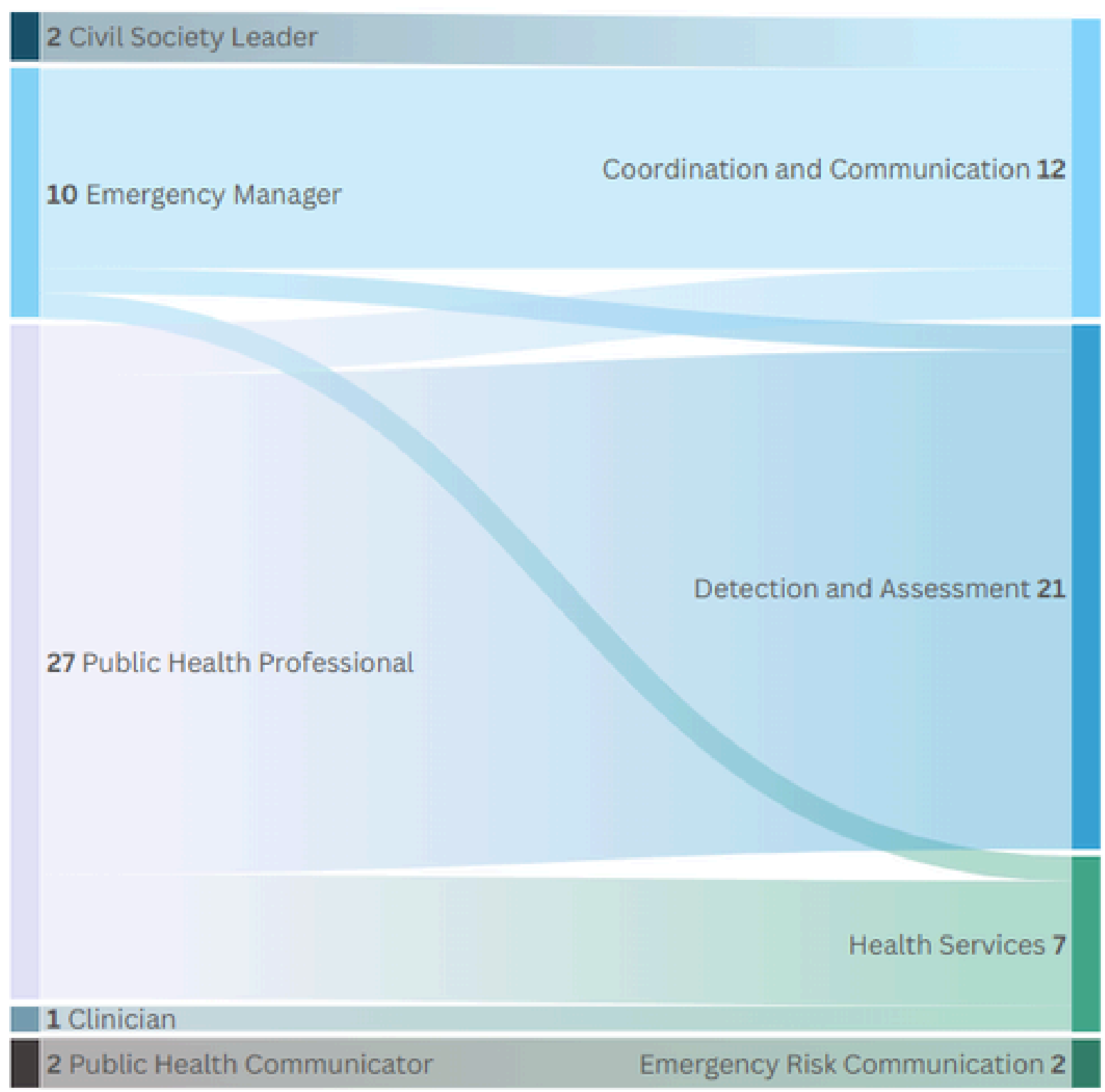

TUNISIA

Population
12.348 M

Income Level
Low Middle

GHS Index Score
31.5

Individual Stakeholders 172

53% Males

47% Females

Stakeholders Organizations 31

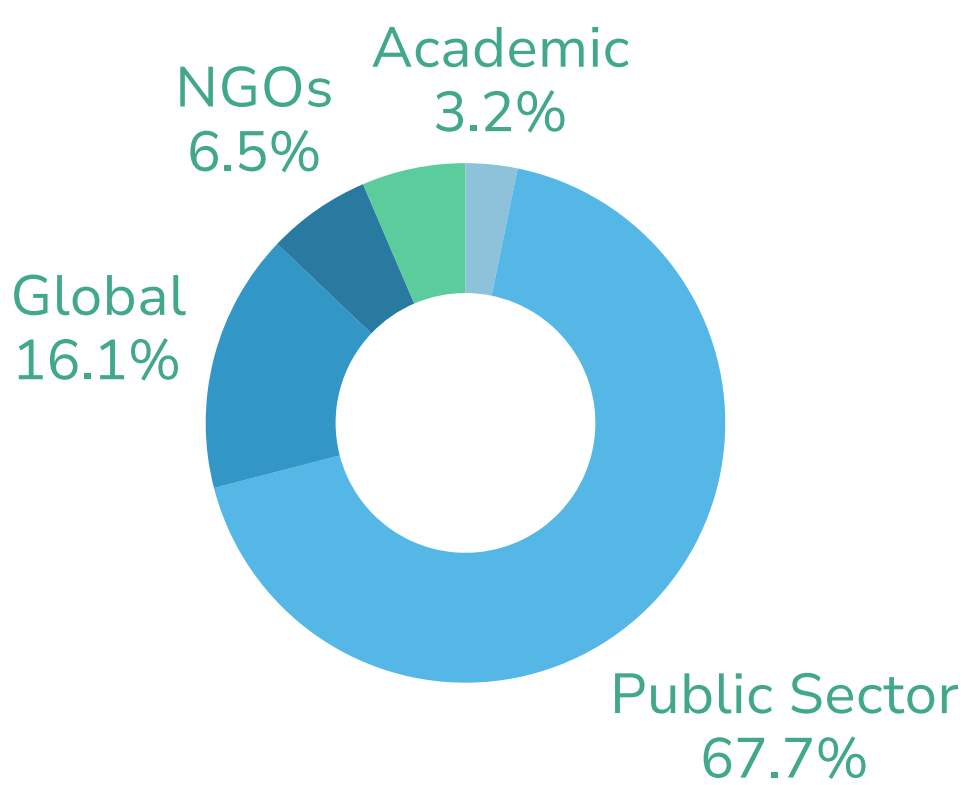

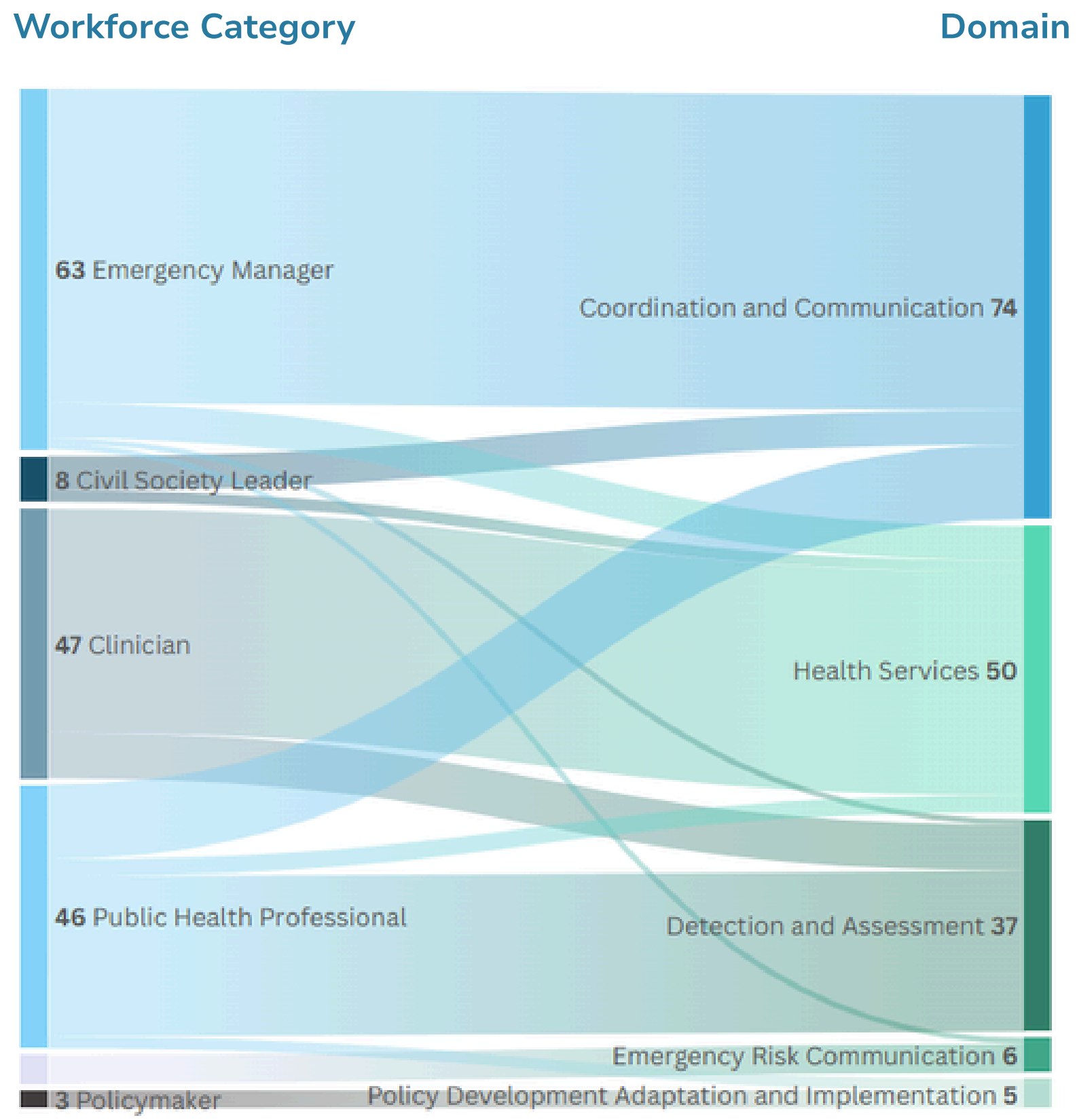

UAE

Population
11.346 M

Income Level
High

GHS Index Score
39.6

Individual Stakeholders 360

55% Males 45% Females

Stakeholders Organizations 92

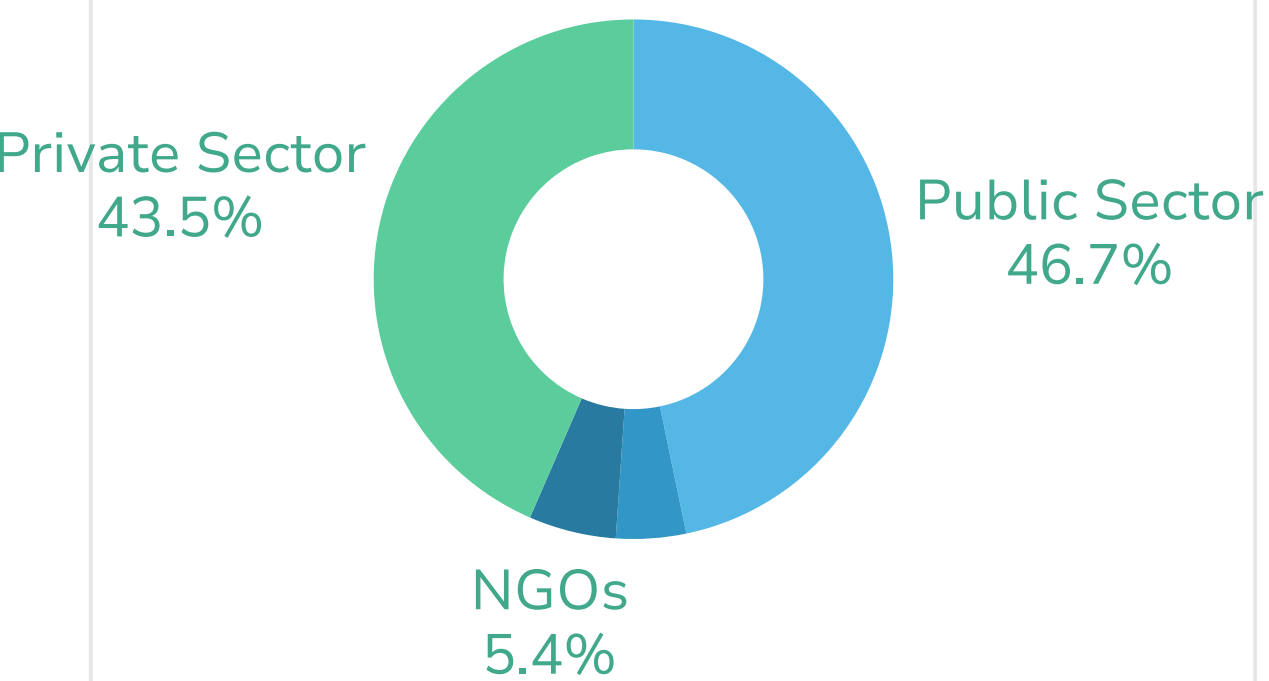

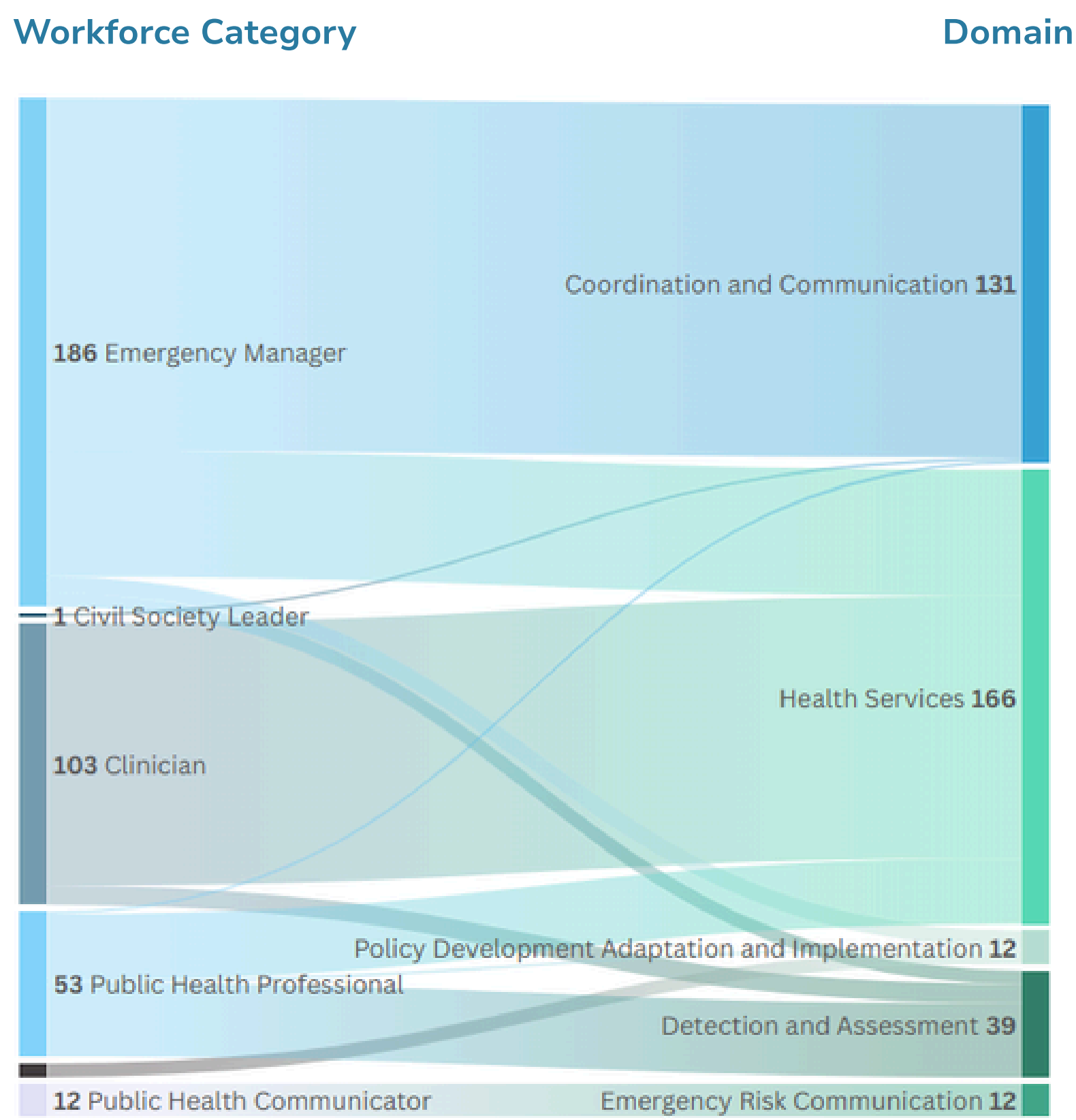

YEMEN

Population
41.773 M

Income Level
Low

GHS Index Score
16.1

Individual Stakeholders 120

83% Males

17% Females

Stakeholders Organizations 33

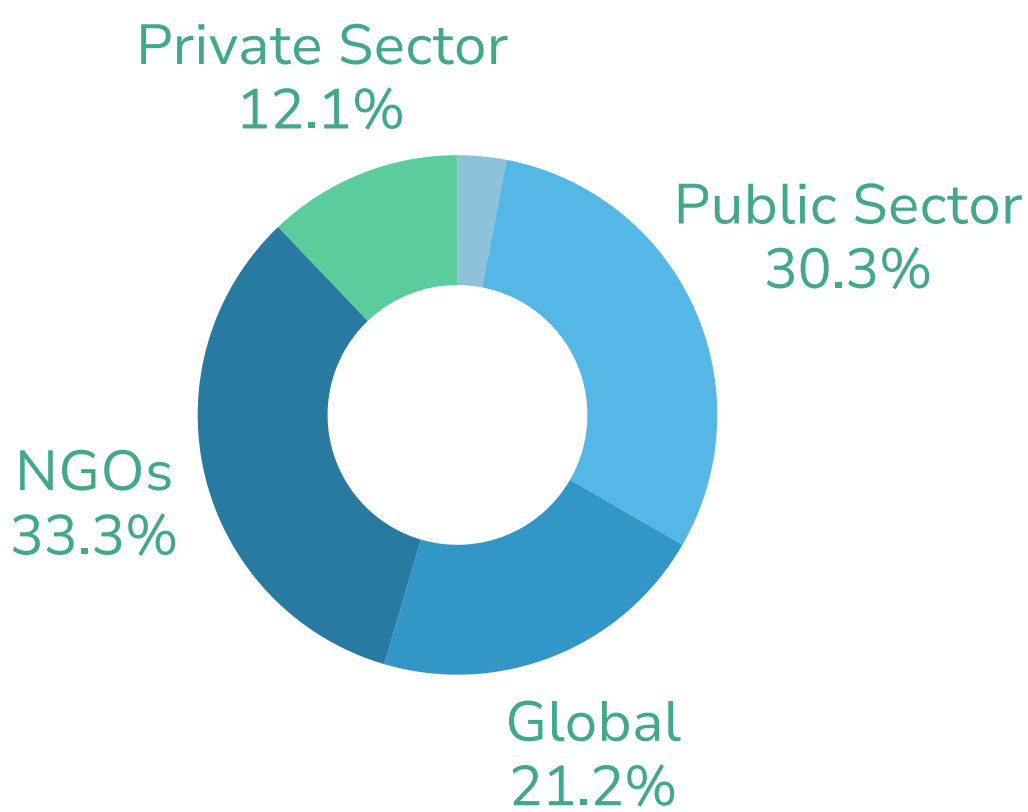

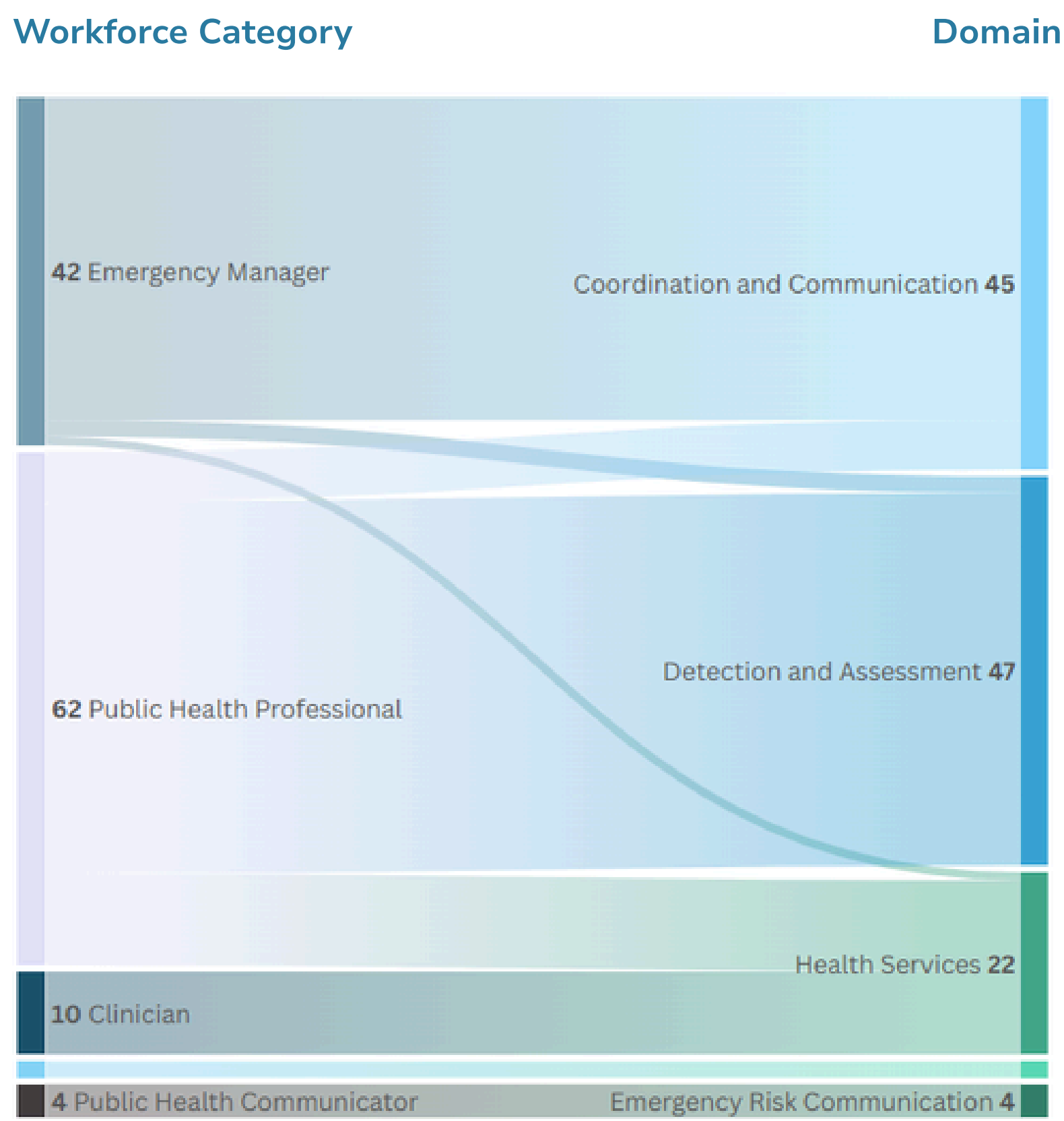

Supplement: Supplementary file 1 [file pgph.0007574.s003.pdf]
