# Supplementary material for: Epidemic and pandemic preparedness in the MENA region: A descriptive analysis of stakeholder distribution, patterns, and regional variation
Source: PLOS Glob Public Health. 2026 Nov 6;6(11):e0007574. doi: 10.1371/journal.pgph.0007574 (PMC13742938; doi:10.1371/journal.pgph.0007574)
Supplement: Supplementary file 2 [file pgph.0007574.s001.docx]

| **Emergency Preparedness and Response** | **Healthcare System Professionals** |
| --- | --- |
| Health emergency preparedness  Communicable disease preparedness  COVID-19 preparedness  Outbreaks and disaster management  COVID-19 response  Infection Control and Prevention | Controlling doctors  Hospital directors  Medical directors  Health specialists  Procurement and management of medical supplies managers  COVID-19 vaccine committee members |
| **International Organizations** | **Local Non-governmental Organizations** |
| UN agencies  World Health Organization  International non-governmental organizations (INGOs | Civil society organizations working in public health and health emergencies |
| **Governmental Agencies** | **Civil Society Actors** |
| Ministries of Public Health  National Public Health Institutes  Syndicates  Public hospitals  Red Cross and Red Crescent Societies  Primary Health Care Centers (PHCs) | UN agencies representatives  UN agencies emergency managers  Civil protection agency leaders |
| **Private Organizations (local)** | **Public Health Professionals** |
| Private hospitals  PHCs  Private health diagnostic centers (laboratories) | Public health agency leaders  Public health legal advisors  Public health emergency managers  Public health preparedness planners  Epidemiologists  Microbiologists  Communicable disease specialists  Infection control experts  Public health veterinarians  Risk communicators  Liaisons with local public health institutes  Health journalists  Health economists  Health information system officers |
| **Governmental Officials** |  |
| Health officials  Heads of healthcare system departments  Liaison with non-governmental hospitals  Public safety officers |  |

Keywords Used for Stakeholder Identification and Mapping
