# Supplementary material for: Epidemic and pandemic preparedness in the MENA region: A descriptive analysis of stakeholder distribution, patterns, and regional variation
Source: PLOS Glob Public Health. 2026 Nov 6;6(11):e0007574. doi: 10.1371/journal.pgph.0007574 (PMC13742938; doi:10.1371/journal.pgph.0007574)
Supplement: Supplementary file 3 [file pgph.0007574.s002.docx]

| **Country** | **Stakeholder-role records (n)** | **Population (M)** | **Records per 100,000 population** |
| --- | --- | --- | --- |
| Bahrain | 193 | 1.643 | **11.75** |
| Palestine | 326 | 5.589 | **5.83** |
| Lebanon | 341 | 5.849 | **5.83** |
| Comoros | 51 | 0.882 | **5.78** |
| Djibouti | 52 | 1.184 | **4.39** |
| Qatar | 102 | 3.115 | **3.27** |
| Emirates | 360 | 11.346 | **3.17** |
| Kuwait | 130 | 5.026 | **2.59** |
| Oman | 140 | 5.494 | **2.55** |
| Libya | 128 | 7.458 | **1.72** |
| Tunisia | 172 | 12.348 | **1.39** |
| Jordan | 147 | 11.520 | **1.28** |
| Somalia | 177 | 19.654 | **0.90** |
| Mauritania | 42 | 5.315 | **0.79** |
| Morocco | 160 | 38.430 | **0.42** |
| Algeria | 187 | 47.435 | **0.39** |
| Sudan | 170 | 51.662 | **0.33** |
| Egypt | 324 | 118.365 | **0.27** |
| Yemen | 120 | 41.773 | **0.29** |
| Saudi Arabia | 94 | 34.566 | **0.27** |
| Iraq | 97 | 47.020 | **0.21** |
| Syria | 42 | 25.620 | **0.16** |

Identified stakeholder-role records by country and population-standardized values
