# Supplementary material for: Epidemic and pandemic preparedness in the MENA region: A descriptive analysis of stakeholder distribution, patterns, and regional variation
Source: PLOS Glob Public Health. 2026 Nov 6;6(11):e0007574. doi: 10.1371/journal.pgph.0007574 (PMC13742938; doi:10.1371/journal.pgph.0007574)
Supplement: Supplementary file 4 [file pgph.0007574.s005.doc]

**STROBE Checklist**

Manuscript: Epidemic and pandemic preparedness in the MENA region: a descriptive analysis of stakeholder distribution, patterns, and regional variation

|  | Item No | Recommendation | Reported on Page(s) |
| --- | --- | --- | --- |
| **Title and abstract** | 1 | (*a*) Indicate the study’s design with a commonly used term in the title or the abstract | p. 2 (Abstract: “cross-sectional stakeholder mapping study”) |
|  |  | (*b*) Provide in the abstract an informative and balanced summary of what was done and what was found | p. 2 |
| Introduction | | |  |
| Background/rationale | 2 | Explain the scientific background and rationale for the investigation being reported | pp. 3-5 |
| Objectives | 3 | State specific objectives, including any prespecified hypotheses | p. 5 (study aim); no prespecified hypotheses |
| Methods | | |  |
| Study design | 4 | Present key elements of study design early in the paper | pp. 5-6 |
| Setting | 5 | Describe the setting, locations, and relevant dates, including periods of recruitment, exposure, follow-up, and data collection | pp. 6-9 (22 MENA countries; searches/data collection during 2023 - 2024) |
| Participants | 6 | (*a*) Give the eligibility criteria, and the sources and methods of selection of participants | pp. 6-9 (stakeholder definition, public-source search, relevance screening and eligibility criteria) |
| Variables | 7 | Clearly define all outcomes, exposures, predictors, potential confounders, and effect modifiers. Give diagnostic criteria, if applicable | pp. 6, 9-10 (preparedness domains, workforce categories, organization, country, income level and gender; no clinical diagnostic criteria) |
| Data sources/ measurement | 8* | For each variable of interest, give sources of data and details of methods of assessment (measurement). Describe comparability of assessment methods if there is more than one group | pp. 7–10 |
| Bias | 9 | Describe any efforts to address potential sources of bias | p. 8 (country-specific adaptation and saturation); pp. 24-25 (visibility bias and classification limitations) |
| Study size | 10 | Explain how the study size was arrived at | p. 8 (country-level data saturation; no fixed target); p. 11 (final N = 3,555) |
| Quantitative variables | 11 | Explain how quantitative variables were handled in the analyses. If applicable, describe which groupings were chosen and why | p. 10 (categorical analyses and four World Bank income groups); no continuous variables were analysed |
| Statistical methods | 12 | (*a*) Describe all statistical methods, including those used to control for confounding | p. 10 (frequencies, percentages and chi-square tests) |
|  |  | (*b*) Describe any methods used to examine subgroups and interactions | p. 10 (stratification by income level); pp. 15-17 (income-level analyses) |
|  |  | (*c*) Explain how missing data were addressed | p. 10 |
|  |  | (*d*) If applicable, describe analytical methods taking account of sampling strategy | N/A - structured public-source stakeholder mapping |
|  |  | (*e*) Describe any sensitivity analyses | N/A |
| Results | | |  |
| Participants | 13* | (a) Report numbers of individuals at each stage of study—eg numbers potentially eligible, examined for eligibility, confirmed eligible, included in the study, completing follow-up, and analysed | pp. 7-9 describe iterative screening; p. 11 reports final N = 3,555. |
|  |  | (b) Give reasons for non-participation at each stage | N/A |
|  |  | (c) Consider use of a flow diagram | N/A |
| Descriptive data | 14* | (a) Give characteristics of study participants (eg demographic, clinical, social) and information on exposures and potential confounders | pp. 11-17; Tables 1-3 |
|  |  | (b) Indicate number of participants with missing data for each variable of interest | p. 10 describes available-data handling; variable-specific missing counts are not separately reported |
| Outcome data | 15* | Report numbers of outcome events or summary measures | pp. 11-17 |
| Main results | 16 | (*a*) Give unadjusted estimates and, if applicable, confounder-adjusted estimates and their precision (eg, 95% confidence interval). Make clear which confounders were adjusted for and why they were included | pp. 11-17; Tables 1-3 |
|  |  | (*b*) Report category boundaries when continuous variables were categorized | N/A |
|  |  | (*c*) If relevant, consider translating estimates of relative risk into absolute risk for a meaningful time period | N/A |
| Other analyses | 17 | Report other analyses done—eg analyses of subgroups and interactions, and sensitivity analyses | pp. 15-17 |
| Discussion | | |  |
| Key results | 18 | Summarise key results with reference to study objectives | p. 18 and pp. 25-26 |
| Limitations | 19 | Discuss limitations of the study, taking into account sources of potential bias or imprecision. Discuss both direction and magnitude of any potential bias | pp. 24-25 |
| Interpretation | 20 | Give a cautious overall interpretation of results considering objectives, limitations, multiplicity of analyses, results from similar studies, and other relevant evidence | pp. 18-26 |
| Generalisability | 21 | Discuss the generalisability (external validity) of the study results | pp. 24-25 |
| Other information | | |  |
| Funding | 22 | Give the source of funding and the role of the funders for the present study and, if applicable, for the original study on which the present article is based | Financial Disclosure adjusted in response to reviewers. |
